# The Smc5/6 Complex Restricts HBV when Localized to ND10 without Inducing an Innate Immune Response and Is Counteracted by the HBV X Protein Shortly after Infection

**DOI:** 10.1371/journal.pone.0169648

**Published:** 2017-01-17

**Authors:** Congrong Niu, Christine M. Livingston, Li Li, Rudolf K. Beran, Stephane Daffis, Dhivya Ramakrishnan, Dara Burdette, Leanne Peiser, Eduardo Salas, Hilario Ramos, Mei Yu, Guofeng Cheng, Michel Strubin, William E. Delaney IV, Simon P. Fletcher

**Affiliations:** 1 Gilead Sciences, Foster City, California, United States of America; 2 Department of Microbiology and Molecular Medicine, University Medical Center (C.M.U.), Geneva, Switzerland; Indiana University, UNITED STATES

## Abstract

The structural maintenance of chromosome 5/6 complex (Smc5/6) is a restriction factor that represses hepatitis B virus (HBV) transcription. HBV counters this restriction by expressing HBV X protein (HBx), which targets Smc5/6 for degradation. However, the mechanism by which Smc5/6 suppresses HBV transcription and how HBx is initially expressed is not known. In this study we characterized viral kinetics and the host response during HBV infection of primary human hepatocytes (PHH) to address these unresolved questions. We determined that Smc5/6 localizes with Nuclear Domain 10 (ND10) in PHH. Co-localization has functional implications since depletion of ND10 structural components alters the nuclear distribution of Smc6 and induces HBV gene expression in the absence of HBx. We also found that HBV infection and replication does not induce a prominent global host transcriptional response in PHH, either shortly after infection when Smc5/6 is present, or at later times post-infection when Smc5/6 has been degraded. Notably, HBV and an HBx-negative virus establish high level infection in PHH without inducing expression of interferon-stimulated genes or production of interferons or other cytokines. Our study also revealed that Smc5/6 is degraded in the majority of infected PHH by the time cccDNA transcription could be detected and that HBx RNA is present in cell culture-derived virus preparations as well as HBV patient plasma. Collectively, these data indicate that Smc5/6 is an intrinsic antiviral restriction factor that suppresses HBV transcription when localized to ND10 without inducing a detectable innate immune response. Our data also suggest that HBx protein may be initially expressed by delivery of extracellular HBx RNA into HBV-infected cells.

## Introduction

Approximately 250 million individuals have chronic hepatitis B (CHB), and more than 780,000 people die each year due to HBV-associated liver diseases, such as cirrhosis and hepatocellular carcinoma (HCC) [1,2]. Multiple nucleos(t)ide analogs as well as interferon-α (IFN-α) are approved for the treatment of CHB, but since these therapies rarely lead to cure [3] there is an urgent need to develop novel antiviral therapies. Therapeutic targeting of the HBV X protein (HBx) is attractive because this viral protein is essential for HBV infection in vivo [4–6] and is required for the initiation and maintenance of viral replication after in vitro infection [7]. Recent work has indicated that HBx plays this key role in the viral lifecycle by maintaining the covalently-closed circular DNA (cccDNA) HBV genome in a transcriptionally active state [7–9]. Pharmaceutical targeting of HBx therefore has the potential to transcriptionally silence cccDNA. This would be an attractive therapeutic response since reducing viral antigen levels may restore effective antiviral immunity and drive patients towards functional cure [10]. Moreover, HBx has been implicated in both the development and progression of HCC [11,12] and so inhibiting HBx function may also have potential as a novel therapeutic approach for the prevention and/or treatment of HBV-related HCC.

We recently determined that cccDNA transcription is inhibited by the structural maintenance of chromosome 5/6 complex (Smc5/6), and that the key function of HBx is to redirect the DDB1 E3 ubiquitin ligase to target this complex for degradation [13]. In this way, HBx alleviates transcriptional repression by Smc5/6 and stimulates HBV gene expression. However, the mechanism by which Smc5/6 restricts HBV transcription and how HBx is first expressed (since it is required for cccDNA transcription) has not been determined. It is also not known whether degradation of Smc5/6 by HBx plays a role in HBV pathogenesis. This is apposite because Smc5/6 has an essential role in maintaining cellular genomic stability and knock-out of both Smc6 and NSMCE2 (a subunit of Smc5/6) are embryonic lethal in mice [14,15], Moreover, loss of Smc5/6 may predispose to genetic errors under conditions of DNA damage [16], and reduced expression of the NSMCE2 subunit is associated with increased cancer incidence in mice [15]. Therefore, while targeting Smc5/6 for degradation stimulates HBV gene expression, it may also contribute to the development and/or progression of HBV-related HCC.

Identifying the spatial relationship between cccDNA, Smc5/6 and other nuclear components may help elucidate the mechanism of HBV restriction by this host complex. Unfortunately, the low copy number of cccDNA together with technical challenges in differentiating it from other HBV nucleic acid species such as relaxed circular DNA (rcDNA), a replicative intermediate, have made it challenging to detect cccDNA in situ. However, chromatin immunoprecipitation (ChIP) studies indicate that Smc5/6 directly interacts with cccDNA [13], suggesting that the HBV genome may co-localize with this complex. Notably, Smc5/6 localizes to Nuclear Domain 10 (ND10) associated with telomeres in immortalized ALT (alternative lengthening of telomeres) cell lines [17]. ND10, also known as PML bodies, are intranuclear complexes comprised of approximately 80 host proteins, including promyelocytic leukemia protein (PML) and speckled protein of 100 kDa (Sp100) [18,19]. ND10 play a role in a variety of cellular homeostatic pathways, including host antiviral defense [18–21]. ND10 components traffic to the incoming genomes of many DNA viruses and restrict transcription from these extrachromosomal viral templates [20–22]. In turn, many DNA viruses encode regulatory proteins that mediate the degradation or disruption of ND10 components [20,21]. It is not known if Smc5/6 localizes to ND10 in non-transformed cells, such as primary human hepatocytes (PHH). Similarly, although HBV DNA has been reported to localize to PML foci in a stably HBV-producing hepatoma-derived cell line [23], the role of ND10 in the viral replication cycle has not been studied in HBV-infected PHH.

Host nuclear viral DNA sensors, such as IFI16, have recently been identified [24]. These pattern recognition receptors (PRRs) recognize pathogen-associated molecular patterns (PAMPs) in virally-infected cells and trigger innate immune responses, including production of IFNs and other cytokines. It is therefore notable that IFN-α treatment can silence HBV transcription and induces similar cccDNA epigenetic changes to those observed with HBx-negative virus (HBVΔX), i.e. when cccDNA is transcriptionally repressed by Smc5/6 [7,25]. Since our previous data suggested that Smc5/6 directly interacts with cccDNA [13], it is possible that this complex senses the HBV genome and indirectly suppress cccDNA transcription by activating the hepatocyte innate immune response. Alternatively, Smc5/6 may inhibit HBV transcription independently of innate immune signaling either by sterically hindering access of host transcription factors and/or RNA polymerase to cccDNA or by directly modulating the epigenetic or topological state of cccDNA [9,26,27]. Since interfering with viral escape from Smc5/6 may identify new therapeutic targets to activate antiviral responses within infected hepatocytes, determining the mechanistic basis of HBV restriction by this complex is an important new area of research.

While various hepatocyte cell lines that are susceptible to HBV infection have been developed [28–30], PHH derived from normal human donors likely represent the most physiologically relevant model for in vitro HBV infection studies. In the present study we report that PML and Sp100 influence the spatial organization of Smc5/6 in the nucleus of PHH, and that association of Smc5/6 to ND10 is important for transcriptional silencing of cccDNA. This study also reveals that HBx RNA is present in cell culture-derived virus preparations as well as CHB patient plasma. Smc5/6 is depleted from the majority of HBV-infected PHH by the time that cccDNA transcription can be detected, suggesting a functional role for this extracellular HBx RNA. Finally, our data suggest that Smc5/6 restriction of cccDNA transcription is not associated with induction of a detectable innate immune response. Collectively, these data indicate that Smc5/6 functions as an intrinsic restriction factor which suppresses HBV when localized to ND10. In addition, the detection of extracellular HBx RNA suggests that HBV employs a novel strategy amongst DNA viruses to counteract restriction by a host factor early after infection.

## Results

### Smc5/6 co-localizes with PML and Sp100 in human hepatocytes

**Fig 1:**
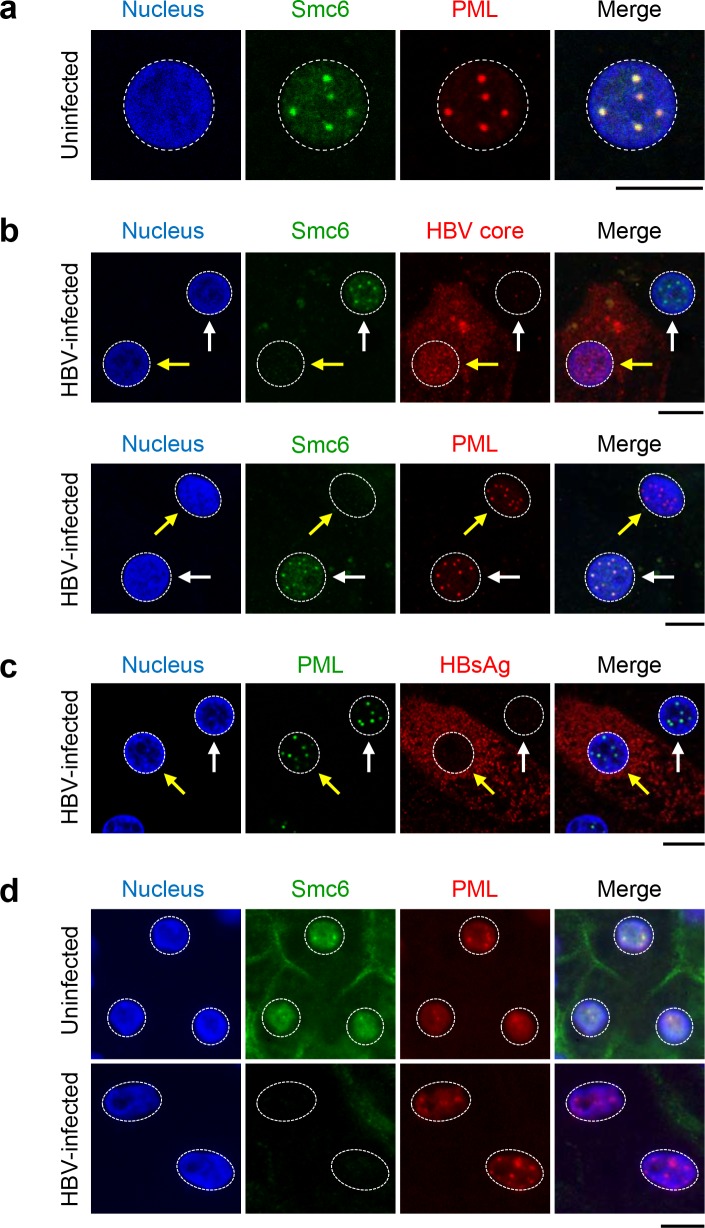
Smc6 co-localizes with PML in uninfected human hepatocytes in vitro and in vivo. (a) Uninfected PHH were stained for Smc6 (green) and PML (red). Nuclei were stained with DAPI (blue). (b, c) PHH were infected with HBV for 13 days. PHH were stained as for (a), and were also stained for HBV core or HBV S antigen (HBsAg) where indicated. Yellow arrows indicate HBV-infected PHH (i.e. HBV core-positive, HBsAg-positive or Smc6-negative), white arrows indicate uninfected cells (i.e. HBV core-negative, HBsAg-negative or Smc6-positive). (a)**-**(c) are representative images from at least n = 3 independent experiments performed with two independent PHH donors. (d) Liver tissue from one representative uninfected (top row) and one HBV-infected (bottom row) humanized mouse were stained as for (a). In the infected animals, the vast majority of human hepatocytes were positive for HBsAg and negative for Smc6 [13]. Comparable images were obtained with liver tissue from additional uninfected (n = 1) and HBV-infected (n = 4) mice. All scale bars represent 10 μm. Nuclei are outlined by white dotted lines in all images.

It has previously been shown that infection of PHH by HBV, but not HBVΔX, leads to degradation of Smc5/6 and that knock-down of Smc6 rescues HBVΔX transcription [13]. Interestingly, confocal imaging from this previous study revealed that Smc6 localized to nuclear foci in uninfected PHH, and that these nuclear substructures were reminiscent of ND10 bodies. In light of this observation, we evaluated whether Smc5 and Smc6 co-localize with components of ND10 in human hepatocytes. First, we determined that Smc6 co-localizes in the nucleus of human hepatocytes with PML, the primary structural component of ND10, both in vitro and in vivo (Fig 1A and 1D). We next confirmed that Smc5 co-localizes with both Smc6 and PML in PHH (S1A Fig). Finally, we demonstrated that both Smc6 and PML co-localize with Sp100, another component of ND10 (S1A Fig). Single cell quantitation revealed >90% of Smc5, Smc6, PML and Sp100 foci co-localized (S1B Fig), confirming that the nuclear Smc5/6 foci in human hepatocytes represent localization to ND10.

### HBV infection does not alter PML and Sp100 levels in human hepatocytes

Many DNA viruses target ND10 components for degradation or disruption [20,21]. We therefore evaluated whether HBx induces selective degradation of Smc5/6 from ND10 or whether these nuclear bodies are altered by HBV infection. By fluorescence microscopy we determined that PML is not depleted in HBV-infected hepatocytes in vitro and in vivo (Fig 1B–1D). In contrast, Smc6 is degraded and disappears from ND10 after HBV infection (Fig 1B and 1D). Consistent with these findings, Western blot analysis revealed that HBV infection reduced levels of Smc6, but not PML or Sp100 (S2A Fig, black vs. grey bars). These data indicate that HBV promotes destruction of the Smc5/6 restriction factor from ND10, but does not alter PML or Sp100 levels in human hepatocytes.

### Depletion of PML and Sp100 alters the nuclear distribution of Smc6 and stimulates HBVΔX transcription

**Fig 2:**
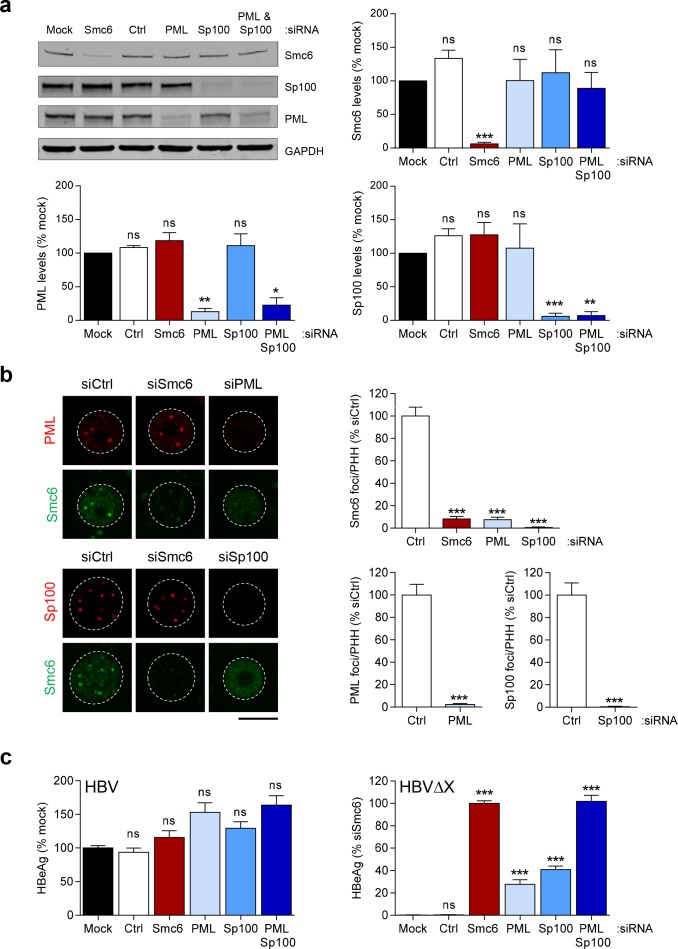
Impact of PML and Sp100 depletion on the nuclear distribution of Smc6 and the HBV replication cycle. (a) Uninfected PHH were transfected with siRNA to the indicated gene(s), a non-targeting control siRNA (Ctrl) or were mock-transfected (mock), and incubated for 13 days. A representative Western blot is displayed on the top left. The blot has been cropped for ease of presentation. All gels were run under the same experimental conditions and full-length blots are presented in S16 Fig. Quantitation of Smc6, PML and Sp100 protein levels (all relative to GAPDH) from n = 4 independent experiments in two independent PHH donors are shown; bar height indicates mean levels expressed as a percentage of mock-transfected cells and the errors bars represent the standard error of the mean (s.e.m.). Statistical significance relative to mock is displayed. (b) Same set-up as (a). Cells were stained for Smc6 (green) and either PML or Sp100 (red). Nuclei were stained with DAPI and outlined by white dotted lines in all images. Representative confocal microscopy images from at least n = 2 independent experiments are shown. Scale bar represents 10 μm. Quantitation of the number of Smc6, PML and Sp100 nuclear foci per PHH expressed as a percentage of the control siRNA is displayed to the right of the representative images. The bar height indicates the mean and the errors bars represent the standard error of the mean; at least n = 44 nuclei were analyzed per siRNA. Statistical significance relative to the control siRNA is displayed. (c) PHH were transfected with siRNA to the indicated gene(s), a non-targeting control siRNA (Ctrl) or were mock-transfected (mock), and incubated for 6 days before infection with wild-type HBV or HBVΔX. HBeAg levels were measured on day 14 post-infection. Mean HBeAg levels ± s.e.m. of n = 6 independent experiments in two independent PHH donors are expressed as a percentage of mock-transfected (wild-type HBV) or siSmc6-transfected (HBVΔX) cells. The mean HBeAg level for mock-transfected wild-type HBV-infected PHH was 1457 ng/mL. Mean HBeAg levels for mock-transfected and siSmc6-transfected HBVΔX-infected PHH were 3 ng/mL and 2199 ng/mL, respectively. Statistical significance relative to mock is displayed. Statistical significance was tested by two-tailed *t*-test (PML and Sp100 foci) or one-way ANOVA with Dunnett's multiple comparison correction (all other comparisons).*p<0.05, **p<0.01 and ***p<0.001, ns: not statistically significant (p>0.05).

Since Smc5/6 localizes to PML and Sp100, we next evaluated whether depletion of these ND10 structural components alters Smc6 levels or localization. Western Blot analysis of uninfected PHH demonstrated that Smc6, PML and Sp100 protein levels were substantially reduced (>75%) after transfection with cognate siRNAs (Fig 2A). Interestingly, while knock-down of PML and/or Sp100 did not significantly alter Smc6 protein levels (Fig 2A), it significantly reduced (>90%) the number of Smc6 foci in PHH (Fig 2B). This reduction in foci number (but not protein levels) suggests that Smc6 is dispersed throughout the nucleus in the absence of PML and Sp100.

In light of this finding, we evaluated whether these ND10 components modulate cccDNA transcription in the presence or absence of HBx. PHH were transfected with siRNAs targeting PML, Sp100 or Smc6, and then infected with HBV or HBVΔX. Consistent with the knock-down in uninfected cells, siRNA treatment substantially (>80%) reduced PML and Sp100 protein levels in both HBV and HBVΔX-infected PHH (S2 Fig). As previously described [13], infection with HBV, but not HBVΔX, induced degradation of Smc6 in PHH (S2 Fig). Treatment with the Smc6 siRNA did not further reduce Smc6 protein levels in HBV-infected PHH, but strongly reduced (>95%) Smc6 protein levels in HBVΔX-infected PHH (S2 Fig). Since Smc5/6 suppresses transcription of all detectable HBV RNAs (S3 Fig), we next performed a series of infection studies with the same siRNAs in which we measured HBeAg levels as a surrogate of cccDNA transcriptional status. HBVΔX transcription was strongly induced by Smc6 knockdown as previously described [13], but was also partially rescued by knockdown of PML or Sp100 (Fig 2C). Strikingly, HBVΔX transcription with knock-down of both PML and Sp100 was comparable to that achieved by knocking-down Smc6 directly (Fig 2C). In contrast, wild-type HBV was not significantly modulated by knockdown of these ND10 structural subunits (Fig 2C). Collectively, these data suggest that PML and Sp100 influence the spatial organization of Smc5/6 in the nucleus, and that localization of Smc5/6 to ND10 is important for silencing of cccDNA transcription in the absence of HBx.

### HBx degrades Smc5/6 early after HBV infection

**Fig 3:**
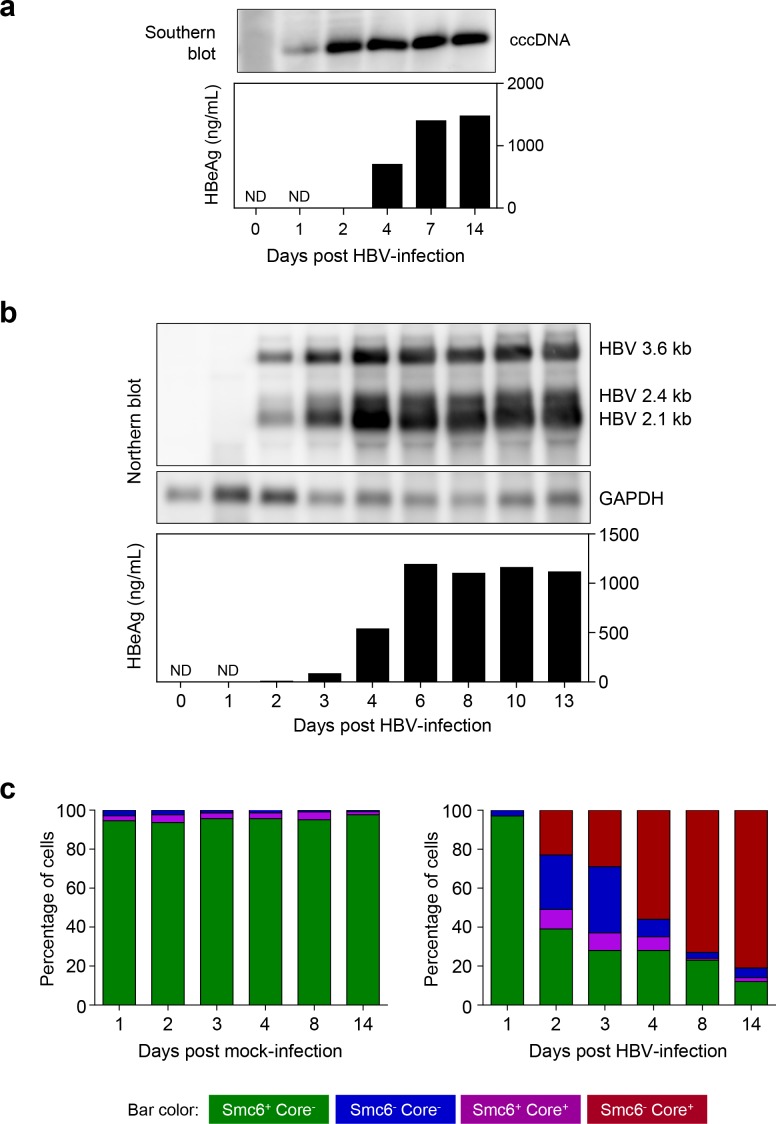
Temporal changes in HBV RNA, HBV core and Smc6 levels after HBV infection. (a) HBV cccDNA and (b) HBV RNA levels were analyzed on the indicated days post HBV-infection by Southern blot and Northern blot, respectively. These blots have been cropped for ease of presentation; full-length blots are presented in S16 Fig. HBeAg levels are shown directly below the corresponding lane in each blot. Cell culture media was changed on days 1, 4, 7 and 10 (Southern blot experiment) and days 1, 3, 6 and 10 (Northern blot experiment). ND: not determined. (c) PHH were mock-infected (left plot) or infected with HBV (right plot). Nuclear-localized HBV core and Smc6 mean fluorescence intensity levels were measured on the indicated days post-infection by confocal microscopy. PHH were stained as in Fig 1B (upper row). Single cell quantitation was performed and each cell defined by nuclear Smc6 and HBV core status. The plots represent the number of cells in each population, expressed as a percentage of the total nuclei analyzed (n≥237 per time-point). The HBV core background was calculated as the mean + 2 x standard deviations of the red channel background signal in mock-infected PHH. The Smc6 background was calculated as the mean—2 x standard deviations of the Smc6 level (green channel) in mock-infected PHH. Values above these limits were considered positive and values below were considered negative. HBV core and Smc6 status of HBV-infected PHH was determined using background levels calculated from time-matched mock-infected controls.

Our previous work indicated that Smc5/6 directly interacts with cccDNA [13], suggesting it might sense the HBV genome and trigger an innate immune response to suppress cccDNA transcription. Therefore, we investigated whether HBV infection induces innate immunity in PHH. However, to guide exploration of the host response to HBV, we first performed a series of studies to characterize the kinetics of viral infection in PHH. In these experiments, cccDNA and HBV RNAs could be detected by day 1 and 2 post-infection, respectively (Fig 3A and 3B). As expected, viral antigen production displayed slower kinetics, with substantial HBeAg (>100 ng/mL) detected from day 4 post-infection (Fig 3A and 3B). Similarly, single cell analysis revealed that the majority of cells were not HBV core-positive until 4 days post-infection, with >80% cells being core positive by day 14 post-infection (Fig 3C, sum of red and purple bars). In contrast, Smc6 was degraded relatively quickly after infection, with >50% of HBV-infected PHH being Smc6-negative by day 2 post-infection (Fig 3C; sum of red and blue bars).

**Fig 4:**
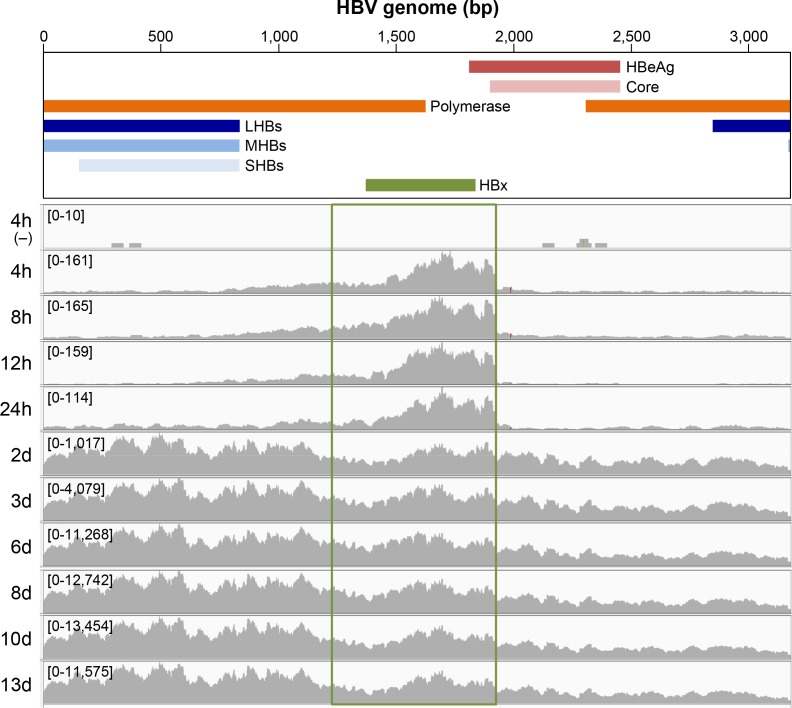
HBx RNA is detected early after HBV infection of PHH. RNA-Seq reads at various time-points after HBV-infection or mock-infection (-) of PHH (donor 1). The study design is outlined in Fig 6A. The x-axis denotes the HBV genome position by reference to the schematic above and the y-axis displays the sequencing coverage of the HBV genome, with the range in parentheses (note the different scale for different time-points). The time-point post-infection is shown adjacent to the y-axis. The green box denotes the HBx transcript region using the canonical transcription start site. h: hour, d: day, bp: base-pair.

The differential kinetics of HBeAg and HBV core production relative to Smc6 degradation (a functional read-out of HBx expression) suggested that HBx may be expressed prior to other viral proteins after HBV infection. This interpretation is consistent with an RNA-Seq analysis which detected low numbers of viral reads that predominantly mapped within the HBx transcript region during the first 24 hours post-infection (Fig 4). This was confirmed in a second PHH donor (S4 Fig) and also with the HBVΔX virus (S5 Fig). Taken together, these data indicate that HBx RNA is detected early after HBV infection, even in the absence of HBx protein.

### HBx RNA is present in cell culture-derived virus preparations and CHB patient plasma

**Fig 5:**
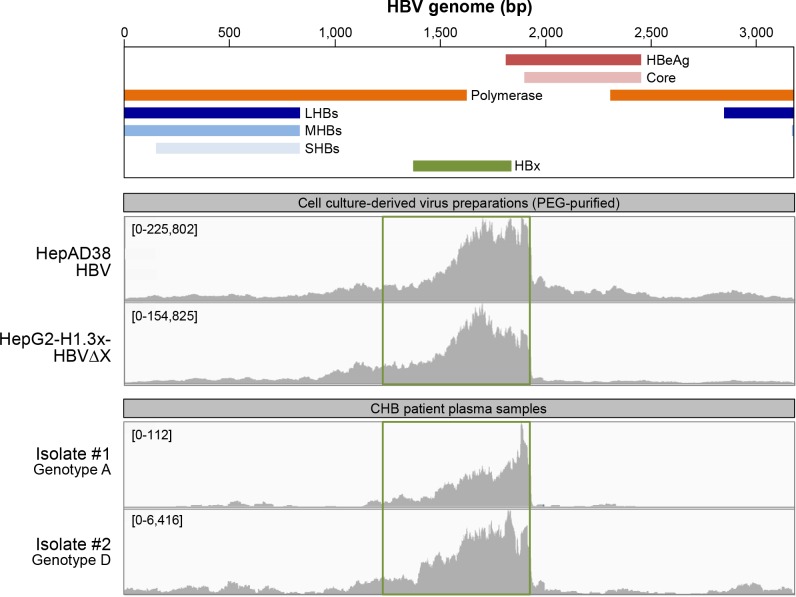
HBx RNA is detected in cell culture-derived virus preparations and CHB patient plasma. RNA-Seq reads in a cell-free virus stocks from HepAD38 and HepG2-H1.3x- cells prepared by PEG precipitation (top) and plasma samples from donors chronically infected with HBV genotype A or D (bottom). Note that HepG2-H1.3x- cells can produce HBVΔX virus in the absence of HBx protein because the HBV genome is integrated into the host genome and is not episomal [7]. HBV DNA levels in the patient isolates were ≥1x10^9^ copies/mL. Of note, RNA-Seq analysis of plasma samples from two additional CHB patients also identified viral RNA reads enriched within the HBx region; however, this data was not included because the maximum coverage of the HBV genome was less than 100. The x-axis denotes the HBV genome position by reference to the schematic above and the y-axis displays the sequencing coverage of the HBV genome, with the range in parentheses (note the different scale for different viruses). The green box denotes the HBx transcript region using the canonical transcription start site. bp: base-pair.

Since HBx RNA was present very early after infection—prior to transcription of the other HBV genes—we next investigated whether HBx RNA is associated with the viral input. RNA-Seq analysis of cell-free virus preparations from HepAD38 cells (which produce wild-type HBV) and HepG2-H1.3x- cells (which produce HBVΔX virus) detected viral reads that predominantly mapped within the HBx transcript region (Fig 5, top). Importantly, viral reads mapping within the HBx transcript region were detected in plasma samples from viremic patients chronically infected with HBV (Fig 5, bottom). It is notable that in all samples there was a lower number of reads mapping to the HBx transcript region before the second AUG in the open reading frame (at nucleotide position 1608) relative to the number of reads mapping to the rest of the HBx RNA. This may indicate a second transcription start site in HBx corresponding to a shorter protein, as has previously been described [31]. Lower numbers of viral reads were also detected in both cell culture and patient plasma samples that mapped across the HBV genome. This is consistent with a recent study which reported that pgRNA is present in HBV-like viral particles in HepAD38 cell supernatant and patient serum [32].

Collectively, these data indicate that HBx RNA and protein is present in PHH shortly after infection. Therefore, if Smc5/6 triggers an innate immune response after detecting cccDNA, activation of hepatocyte antiviral immunity would be expected to be limited to the first few days after infection prior to accumulation of HBx protein. Accordingly, we next performed a series of studies to comprehensively characterize the early host response to infection.

### HBV infection does not substantially alter the host transcriptome nor induce ISG expression in PHH

We first conducted a pilot study in highly infected PHH from a single donor (S6 Fig). To assess potential changes in gene expression between HBV-infected and mock-infected cells, RNA-Seq analysis was performed at ten time-points during the two week infection period. Principal component analysis (PCA) of the transcriptome data revealed close proximity between HBV-infected and time-matched mock-infected PHH, indicating a comparable temporal transcriptional profile in HBV-infected and mock-infected PHH (S6C Fig). However, there was a substantial temporal shift in the host transcriptome in both mock-infected and HBV-infected cells, likely associated to cell seeding and/or DMSO treatment, which stabilized several days post-infection (S6C Fig). These changes were primarily driven by up-regulation of genes associated with lipid and bile acid metabolism and down-regulation of genes associated with protein synthesis (S7 and S8 Figs). Most notably, culturing of the PHH prominently induced the FXR/RXR/LXR activation pathways (S7 Fig). Importantly, genes expressed in mature hepatocytes, such as *albumin* (*ALB*), *NTCP* (*SLC10A1*), *HNF-4α* (*HNF4A*) and *CK18* (*KRT18*), had either stable or increased expression over time (S9 Fig), indicating that the PHH retained characteristics of hepatocytes and were not de-differentiating under the culture conditions. This is consistent with the permissiveness of PHH to HBV infection up to 9 days post-seeding (S10 Fig). Furthermore, cell cycle-related transcriptional pathways were not induced (S7 Fig), indicating that PHH remain in the G_0_ stage of the cell cycle (i.e. are quiescent) after seeding in high (2%) DMSO. Overall, under these experimental conditions, high level HBV infection of PHH had little to no impact on the host transcriptome. However, since the substantial time-dependent changes in the PHH transcriptome may have reduced the sensitivity of this initial analysis in the first few days post-infection, subsequent studies evaluating transcriptional responses shortly after infection were performed in PHH that were not infected until at least 4 days post-seeding.

**Fig 6:**
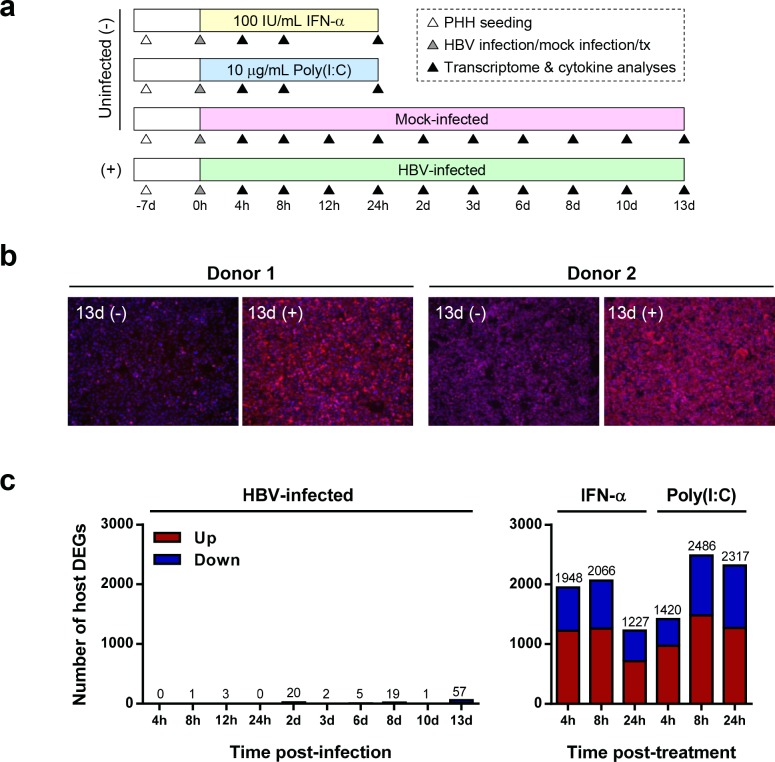
HBV infection does not result in a prominent global transcriptional response in PHH. (a) Schematic summarizing the design of the study with two independent PHH donors (donors 1 and 2). Duplicate samples for each donor were analyzed on day 13 post-infection, with single samples per donor at other time-points. Cell culture media was changed on days 1, 3, 6, 8 and 10. tx: treatment, d: day, h: hour. (b) Immunofluorescence staining of HBV core (red) and nuclei (blue) at 13 days (d) post-infection with HBV (+) or mock (-). (c) Number of differentially expressed genes (DEGs) relative to time-matched mock-infected control for PHH infected with HBV (left panel) or treated with IFN-α or poly(I:C) (right panel) (red, over-expressed; blue, under-expressed). A total of 20,396 genes were analyzed. All DEGs had an absolute mean fold-change >2 and a false discovery rate (FDR) <0.05, and passed a low-expression filter.

**Fig 7:**
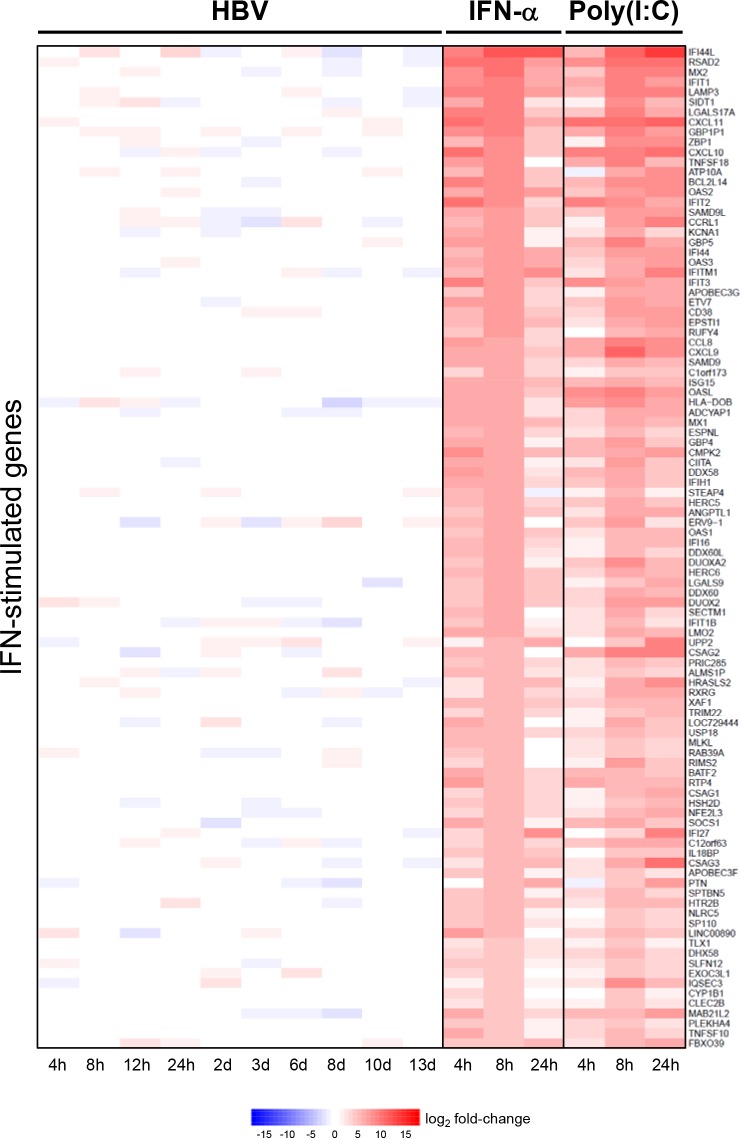
HBV infection does not induce ISG expression in PHH. Expression of top IFN-induced genes in PHH infected with HBV or treated with either IFN-α or poly(I:C) plotted by mean log_2_ fold-change of two independent donors. Heatmap columns represent comparisons relative to time-matched mock-infected control. Rows represent individual genes; over-expression (red) and under-expression (blue) indicated by scale bar for log_2_ fold change values. All genes (n = 100) had a mean fold-change >2 and a false discovery rate (FDR) <0.05 at 8 hours post-treatment with IFN-α relative to the time-matched mock-infected control, and passed a low-expression filter. None of these genes were identified as DEGs at any time post-infection with HBV (S2 Table).

We next performed a study in two independent PHH donors as summarized in Fig 6A. High level infection (~80–90% PHH infected) at day 13 post-infection was confirmed by HBV core antigen staining (Fig 6B). As previously described, transcriptome analysis demonstrated that a substantial increase of total HBV RNA was detected as early as day 2 post-infection (Fig 4, S4 Fig), consistent with the aforementioned Northern blot analysis (Fig 3B). However, HBV infection had only a very modest impact on the PHH transcriptome, with less than 60 of the 20,396 genes analyzed being differentially expressed (FDR<0.05, fold-change >2) at any one time-point post-infection (Fig 6C, S1 Table). Furthermore, higher HBV RNA levels did not correlate with increased number of differentially expressed genes (DEGs), indicating that there is no prominent or consistent transcriptional signature associated with HBV infection in PHH. We confirmed that the PHH can respond to innate immune stimuli by characterizing the transcriptional response of uninfected cells to treatment with IFN-α and poly(I:C) (a TLR3 agonist). As expected, these agents substantially modulated the PHH transcriptome, with >2000 DEGs at 8 hours post-treatment (Fig 6C), including a large number of interferon-stimulated genes (ISGs) (Fig 7). Sendai virus (a RIG-I agonist) also strongly induced ISG expression in the PHH (S11 Fig). In contrast, HBV infection did not induce coordinated expression of ISGs in either donor, and no ISGs were identified as DEGs at any time point post-infection (Fig 7, S2 Table). Notably, all PHH samples had very low sequence counts (range: 0–30 counts from ~30 million total reads per sample) of *STING* (*TMEM173*), an adaptor protein of an innate immune receptor (cGAS) that senses intracellular DNA. This is consistent with a recent study which reported that neither murine or human hepatocytes express STING [33].

### HBV infection does not induce IFNs or other cytokines in PHH

**Fig 8:**
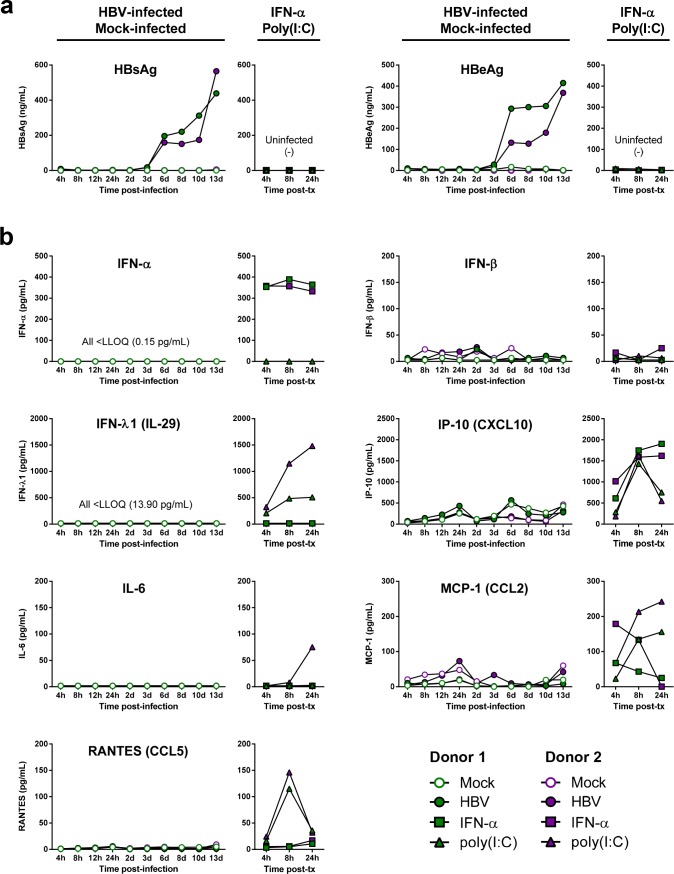
HBV infection does not induce IFNs or other cytokines in PHH. Levels of secreted (a) HBeAg and HBsAg and (b) cytokines in two independent PHH donors infected with HBV or mock-infected (left panels), or treated with either IFN-α or poly(I:C) (right panels). IP-10 (CXCL10) levels at 8 hours post-treatment with poly(I:C) and both 8 and 24 hours post-treatment with IFN-α were greater than the upper limit of quantitation (ULOQ; 1347 pg/mL), and were extrapolated from the standard curve.

To complement the transcriptomic analysis, we evaluated supernatant HBeAg, HBsAg and cytokine levels in the same experiment. In line with the previous study (Fig 3A and 3B), HBV antigen production displayed delayed kinetics relative to HBV RNAs, with substantial HBeAg and HBsAg levels (>100 ng/mL) detected from day 6 post-infection (Fig 8A). Consistent with the transcriptomic analysis, HBV infection did not induce production of IFNs or other cytokines at any time post-infection (Fig 8B, S3 and S4 Tables). In contrast, poly(I:C) rapidly induced IFN-λ1 (IL-29) production, and both poly(I:C) and IFN-α stimulated production of the IFN-inducible chemokine IP-10 (Fig 8B). Various other cytokines were also induced by poly(I:C) and/or IFN-α treatment of PHH (Fig 8B, S3 and S4 Tables). Altogether, these data suggest that Smc5/6 does not trigger a detectable innate immune response upon HBV infection prior to being targeted for degradation by HBx.

### HBVΔX infection does not induce IFNs or ISG expression in PHH

**Fig 9:**
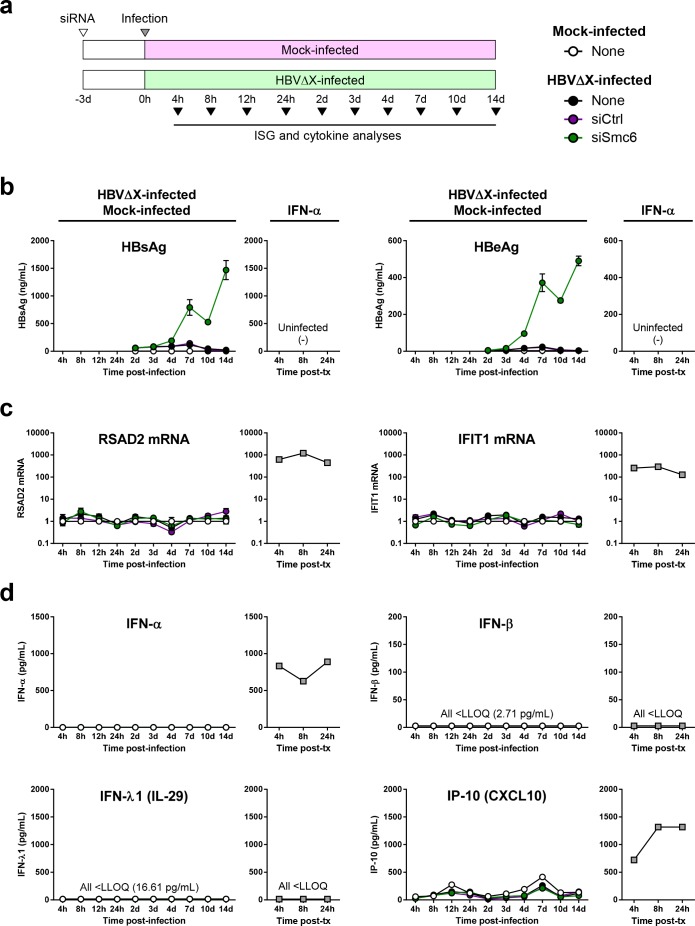
HBVΔX infection does not induce IFNs or ISG expression in PHH. (a) Schematic summarizing the design of the study. PHH were seeded at day (d) -4. Triplicate samples were analyzed at each time-point; samples were pooled prior to luminex analysis. Cell culture media was changed on days 1, 3, 7 and 10. Levels of (b) HBeAg and HBsAg, (c) ISGs and (d) cytokines from PHH infected with HBVΔX or mock-infected (left panels, circles) or in uninfected PHH treated with 100 IU/mL IFN-α (right panels, grey squares). For (b) and (c), the circles and squares indicate the mean, and the errors bars represent the standard error of the mean. The HBVΔX and mock-infected PHH were mock-transfected (none) or transfected with the indicated siRNA, as shown in the legend in (a). RNA levels were expressed as fold-change relative to the time-matched, mock-infected control with no siRNA. HBeAg and HBsAg levels were not measured at 4, 8, 12 or 24 hours (h) post-infection for HBVΔX-infected, mock-infected or IFN-treated PHH. IP-10 levels at 8 and 24 hours post-treatment with IFN-α were >ULOQ (1317 pg/mL), and were extrapolated from the standard curve.

To directly determine whether transcriptional silencing of cccDNA via Smc5/6 is associated with induction of innate immunity, we next studied the PHH response to infection with HBVΔX (Fig 9A). We first demonstrated that there were little to no viral antigens produced by PHH infected with HBVΔX (Fig 9B, none), consistent with cccDNA being largely transcriptionally silent in the absence of HBx. As previously reported [13], HBV antigen levels were restored by Smc6 knock-down, but not with a non-targeting control siRNA (Fig 9B, siSmc6 and siCtrl). The substantial antigens levels (>400 ng/mL) induced by HBVΔX-infected PHH treated with siSmc6 were consistent with high level infection. In a separate study, the same siRNAs were used to demonstrate high level infection (~80–90% PHH infected) with HBVΔX by HBV core antigen staining (S12 Fig).

It has previously been shown that HBVΔX infection leads to establishment of cccDNA but not degradation of Smc5/6 [7,9,13]. Therefore, if Smc5/6 triggers an innate immune response in hepatocytes after detecting cccDNA, it is expected that there would be prolonged activation of antiviral immunity after HBVΔX infection. However, HBVΔX infection did not induce the expression of two prototypic ISGs, *RSAD2* (Viperin) and *IFIT1* (ISG56), at any time point post-infection when cccDNA was transcriptionally silent (Fig 9C, none or siCtrl). HBVΔX infection also did not induce coordinated ISG expression when cccDNA transcription was rescued by knock-down of Smc6 (Fig 9C, siSmc6). The lack of ISG expression at day 13 post-infection with HBVΔX was confirmed using a second PHH donor (S13 Fig). HBVΔX also did not induce the production of IFNs or other cytokines in the presence or absence of Smc6 knock-down (Fig 9D, S5 Table). In contrast, IFN-α treatment strongly induced the expression of ISGs as well as production of IP-10 and various other cytokines (Fig 9C and 9D, S5 Table). Consistent with the lack of host response to HBV, these HBVΔX infection data indicate that transcriptional suppression of cccDNA by Smc5/6 is not associated with induction of a detectable innate immune response.

## Discussion

While the hepatocyte innate immune response to HBV is poorly understood [34], it was recently demonstrated that HBV cccDNA is transcriptionally silenced by the Smc5/6 complex when HBx is not present [13,35]. However, it is not known how Smc5/6 suppresses viral gene expression or whether depletion of this complex plays a role in evasion of innate immunity or HBV pathogenesis. In addition, it is not understood how HBx is initially expressed if it is required to alleviate transcriptional suppression by Smc5/6. In the current study we used PHH, a physiologically relevant system for in vitro studies, to characterize viral kinetics and the host response during the early stages of HBV infection in order to address these unresolved questions.

### Smc5/6 localization to ND10 is important for cccDNA restriction

We first demonstrated that Smc5/6 co-localizes with PML and Sp100 in ND10 in hepatocyte nuclei in the absence of HBV infection. Previously it has been shown that Smc5/6 is recruited to ND10 associated with telomeres in ALT cancer cells [17]. In these cells, Smc5/6 localization to ND10 is cell cycle-regulated and is required for telomere elongation [17]. In contrast to ALT cells, PHH are quiescent (i.e. in G_0_), and therefore Smc5/6 localization to ND10 in these non-dividing hepatocytes is likely not related to a role in telomere maintenance via the ALT pathway. Interestingly, in contrast to certain DNA viruses (e.g. HSV-1) [20,21], HBV infection did not induce degradation of ND10 structural components PML and Sp100. Instead, our data indicate that HBx promotes degradation of Smc5/6 from ND10 during HBV infection, whereas PML and Sp100 levels are unaffected. Notably, depletion of both PML and Sp100 altered the nuclear distribution of Smc6 and induced HBV transcription in the absence of HBx, suggesting that the association of Smc5/6 to ND10 is important for transcriptional silencing of cccDNA. In contrast, our data indicate that neither PML nor Sp100 are required for HBV transcription in the presence of HBx. This suggests that these ND10 components do not play a role in restricting (or stimulating) cccDNA transcription when HBx promotes degradation of Smc5/6, but instead act as a scaffold complex promoting Smc5/6 localization and function.

ND10 components can cooperatively repress transcription of various DNA viruses [36,37], which parallels the observation that simultaneous knock-down of both PML and Sp100 gave the greatest rescue of HBVΔX. However, it is not clear why depletion of both PML and Sp100 was required for maximal induction of HBVΔX transcription, since depletion of each factor individually substantially altered the nuclear distribution of Smc6. Since Smc6 levels were not reduced by PML and Sp100 knock-down, it is possible that some fraction of Smc5/6 is still associated with cccDNA in the absence of either one of these ND10 components, and that only simultaneous knock-down fully prevents association of Smc5/6 with cccDNA. Alternatively, although our previous data indicate that Smc5/6 associates with cccDNA, suggesting a direct mechanism of transcriptional inhibition [13], it is possible that an additional factor also plays a role in suppressing HBV transcription in the absence of HBx. In such a scenario, depletion of Smc5/6, PML or Sp100 may alter the distribution, levels or function of this hypothetical restriction factor, preventing association with cccDNA and resulting in rescue of HBVΔX transcription. Development of a sensitive method to isolate and characterize cccDNA will be required to evaluate these various hypotheses.

Collectively our data suggest that association of Smc5/6 to ND10 is important for suppressing cccDNA transcription. Given that ND10 components traffic to the incoming genomes of many DNA viruses [20–22], it is tempting to speculate that ND10 play an important role in HBV restriction by recruiting Smc5/6 to cccDNA (S14 Fig). It will therefore be interesting to determine whether Smc5/6 plays a role in restriction of other DNA viral genomes that associate with ND10.

### HBx RNA is present in cell culture-derived virus preparations and CHB patient plasma

Viral proteins that counteract host restriction factors are typically packaged within the virus particle or are expressed shortly after infection. However, it has not been established whether HBx protein is contained in the HBV virion, whether the HBx gene is transcribed early after infection from newly formed cccDNA or whether HBV employs an alternative strategy. RNA-Seq analysis revealed that HBx RNA can be detected very early after infection of PHH, prior to depletion of Smc6 and transcription of the other HBV genes. Unexpectedly, further RNA-Seq studies demonstrated that HBx RNA can be detected in cell culture-derived virus preparations as well as in patient plasma samples. It is unlikely that these data are a result of viral DNA contamination since the RNA samples were treated with DNase I and selected with poly(dT) prior to sequencing. This is underscored by analysis of simulated sequences from host intergenic regions which showed no evidence of contaminating DNA. Furthermore, analysis by skewness metrics did not show any evidence of 3'-end bias, suggesting the HBx reads are not derived from pgRNA encapsidated in virus-like particles [32]. These data therefore suggest that HBx RNA is present in viral particles, subviral particles or microvesicles, and provide a potential explanation for how HBx protein is initially expressed in HBV-infected cells. However, while the HBx RNA detected by RNA-Seq must have a poly(A) tail as a consequence of the poly(dT) selection step, it is not known whether this RNA is also 5'-capped. It is also remains to be proven that the extracellular HBx RNA is delivered into the cytoplasm of infected cells and can produce functional HBx protein. In the meantime, a functional role for this extracellular HBx RNA is suggested by the observation that Smc5/6 is degraded in the majority of HBV-infected PHH by the time transcription of viral genes from cccDNA can be detected. Future studies will be focused on determining the origin and nature of this HBx RNA.

### Smc5/6 is an intrinsic antiviral restriction factor

Previous ChIP studies indicated that Smc5/6 directly interacts with cccDNA [13], suggesting that this complex might sense the HBV genome and trigger an innate immune response. However, data from the current study in PHH demonstrated that both HBV and HBVΔX establish high level infection in PHH (~80–90% infected cells) without inducing an ISG or cytokine response, even within the first 24 hours post-infection. It is important to note that, although not all PHH were infected with HBV, the paracrine effect of any cytokines produced by virus-sensing cells would be expected to induce a transcriptional response in uninfected cells. This is illustrated by the strong induction of a large number of ISGs in uninfected PHH by IFN-α treatment. Therefore, the lack of ISG induction by HBV and HBVΔX infection likely does not reflect a lack of sensitivity to detect an innate immune response in this experimental system.

Since Smc5/6 is constitutively expressed in human hepatocytes [13], our data suggest that the function of this complex is analogous to the "intrinsic" antiviral factors that recognize specific components of HIV-1 and directly target the virus without inducing an innate immune response (e.g. APOBEC3G, SAMHD1) [38,39]. Another parallel is that both HBV and HIV-1 have evolved accessory proteins that hijack host ubiquitin ligases and target these restriction factors for degradation [13,39]. However, Smc5/6 is unusual in that it also has important (although poorly defined) roles in maintaining host genomic stability [40,41]. The dual role Smc5/6 plays in host defense and genome maintenance is reminiscent of the DNA damage response (DDR), which functions to preserve host genome integrity as well as defend cells against DNA virus infection [42]. It is also notable that there are close parallels between the DDR to viral DNA and cellular genome breaks [42], indicating that common molecular mechanisms can regulate host genomic integrity and restrict DNA viruses in the nucleus. Since detection of the HBV genome by Smc5/6 does not trigger a detectable innate immune response, our data suggests that the antiviral function of this complex is likely mechanistically related to its role in cellular genome maintenance.

### Implications for the hepatocyte innate immune response to HBV

It has previously been proposed that HBV passively evades or actively inhibits innate immune sensing in hepatocytes [43,44]. While our data does not directly address these two possibilities, it is striking that we were not able to detect an innate immune response in PHH at any time post-infection with HBV or HBVΔX. Moreover, we detected little to no STING expression in PHH, consistent with a recent study which reported that deficiency of this adaptor protein prevents functional DNA sensing in hepatocytes, thereby facilitating HBV infection [33].

Importantly, our data is consistent with a seminal study in chimpanzees acutely infected with HBV [45], together with a recent study in woodchucks acutely infected with woodchuck hepatitis virus (WHV) [46], which indicated that HBV and related hepadnaviruses do not induce an intrahepatic innate immune response. These studies are also in line with the lack of elevated type I IFN levels in the serum of patients with acute HBV infection [47]. In contrast, recent in vitro studies have suggested that HBV can induce a modest IFN response in human hepatocytes [30,48–51]. However, in many of these studies the response of human hepatocytes to HBV was analyzed in the context of high levels of physiologically irrelevant cells (e.g. mouse stromal fibroblasts or mouse innate immune cells) or where additional cell types were present at non-physiologically relevant levels (e.g. cholangiocyte-like cells). In addition to substantial differences in the experimental systems studied, the discrepancy between our data and these other in vitro studies may be explained, at least in part, by the fact that our PHH prior to culture contained ≥85% hepatocytes and only low numbers of non-parenchymal cells (NPCs) (S15 Fig). Notably, there was no positive staining for CD68 (a Kupffer cell marker) by flow cytometry in these PHH. While CD68 mRNA could be detected in the PHH shortly after seeding, the levels were strongly decreased after several days in culture (S9 Fig), indicating that the high DMSO assay conditions further reduced the numbers of NPCs prior to HBV infection. Therefore, our study was not intended to address whether NPCs can be activated by HBV, as has previously been reported [52,53]. It is also important to note that in addition to not differentiating passive evasion versus active inhibition of innate immunity by HBV, our study does not address whether there are any viral genotype differences in hepatocyte innate immune response. Furthermore, although we evaluated host response to HBV infection in PHH from multiple independent donors and demonstrated that these PHH are responsive to relevant innate immune stimuli, it is possible that genetic and/or environmental factors may have influenced the response of these PHH to HBV infection. Future studies will focus on further characterizing the interaction between HBV and innate immunity in PHH.

### Potential role of Smc5/6 depletion in HBV pathogenesis

Another notable finding from our study was that HBV transcription and protein production as well as assembly, maturation and secretion of virions and subviral particles can occur without substantial modulation of the host transcriptome. Importantly, these in vitro data are consistent with the absence of virus-associated intrahepatic transcriptional signatures in chimpanzees and woodchucks acutely infected with HBV and WHV, respectively [45,46]. Moreover, these data also indicate that degradation of Smc5/6 by HBx does not substantially alter gene expression in HBV-infected PHH. This has two important implications. Firstly, it suggests that HBx does not strongly activate cellular transcription pathways in the context of natural infection, and instead supports the notion that Smc5/6 transcriptionally suppresses episomal (i.e. HBV cccDNA) but not genomic DNA [13]. Secondly, the lack of a cellular stress response to HBV infection indicates that Smc5/6 is not necessary for hepatocyte viability under the conditions evaluated. Although knock-out of both Smc6 and NSMCE2 are embryonic lethal in mice [14,15], it is important to note that our PHH transcriptome data are consistent with the observation that over-expression of HBx does not have deleterious effects on non-dividing cells [54]. In contrast, over-expression of HBx induces genomic instability in dividing cells, with over-expression of HBx and depletion of Smc5/6 both being associated with lagging chromosomes during cell division [54,55]. Moreover, unlike the PHH in vitro infection system, chronic HBV infection is characterized by cycles of liver damage and regeneration as well as widespread DNA damage over long periods of time [56]. Under these conditions, the loss of Smc5/6 may play a role in disease pathogenesis by contributing to the development and/or progression of HBV-related HCC [15,16].

While our data strongly suggests that HBV infection does not induce a significant transcriptional response in PHH, it is important to note that there was a substantial temporal shift in the host transcriptome in the absence of HBV infection. This is in line with recent studies in PHH as well as primary rat hepatocytes [57,58]. Notably, we showed that seeding and/or DMSO treatment significantly induced lipid and bile acid metabolism pathways, with *CYP7A1* expression in particular being strongly up-regulated (S9 Fig). It has previously been reported that these transcriptional pathways are associated with HBV infection in the humanized mouse model [59]. Although the PHH transcriptome changes stabilized after several days in culture, and delaying infection for several days substantially reduced these early changes in the host transcriptome after infection, gene expression changes related to seeding and/or DMSO treatment may still have confounded identification of a modest metabolic signal induced by HBV infection in PHH. In addition, although only a small proportion of PHH (~10–20% cells) were not infected with HBV, it is possible that our transcriptome analyses failed to identify virus-induced changes in the expression of low abundance genes.

In summary, the current study expands our understanding of key host-virus interactions that contribute to HBV persistence (and potentially pathogenesis), and underscores the potential of HBx as a novel target for the treatment of chronic HBV infection.

## Methods

### Ethics statement

Primary human hepatocytes (PHH) isolated from deceased donor livers were purchased from Life Technologies (Grand Island, NY) and BioreclamationIVT (Westbury, NY). Consent was obtained from the donor or the donor's legal next of kin for use of the tissue and its derivatives for research purposes using IRB-approved authorizations. Plasma from CHB patients was purchased from Proteogenex (Culver City, CA). Consent was obtained from the donor for use of the sample for research purposes using IRB-approved authorizations. All animal work was performed by PhoenixBio Co, Ltd (Higashi-Hiroshima City, Japan). The animal protocol and all procedures involving mice were approved by the Animal Welfare Committee of PhoenixBio Co, Ltd (Protocol Number: PBC-HI14-028) and adhered to the Guide for the Care and Use of Laboratory Animals. All surgery was performed under isoflurane anesthesia, and all efforts were made to minimize suffering.

### Reagents

Recombinant human IFN-α2a was from PBL Assay Science (Piscataway, NJ). High molecular weight polyinosinic-polycytidylic acid (poly(I:C)) was from InvivoGen (San Diego, CA). Sendai virus (SeV, Cantell strain) was from Charles River Laboratories (North Franklin, CT). The HepAD38 and the HepG2-H1.3x- stable cell lines have been previously described [7,60] and were obtained from Avid Therapeutics and Ulrike Protzer, respectively. All siRNAs were obtained from Dharmacon (Lafayette, CO). The Smc6 (siSmc6), DDB1 (siDDB1) and control (siCtrl1) siRNAs have been previously described [13]. The PML and Sp100 siRNA sequences are as follows; siPML: 5’-GCAAAGAGUCGGCCGACUU-3’; siSp100: 5'-UGACAACCCUUUAGAAUCA-3'. The PML and Sp100 siRNAs were designed to the N-terminus of the mRNAs in order to target all isoforms of these proteins.

### HBV virion production and PHH infection

Production of wild-type HBV virions from HepAD38 cells was performed as previously described [13]. HBVΔX virions were produced from HepG2-H1.3x- stable cell line using the same protocol. For infection studies, cryopreserved PHH were thawed and then seeded in cell plating medium (Life Technologies) at 50,000–65,000 cells per well in 96-well collagen-coated plates (Life Technologies). The PHH donors used in this study were selected based on the ability of the cells to plate and maintain high viability in culture. At 6 hours post-plating, the media was replaced with maintenance medium (Life Technologies) containing 2% DMSO and 2% FBS, and the cells were then incubated at 37°C in a humidified 5% CO_2_ incubator. Cells were infected with either HepAD38 wild-type HBV virions or HepG2-H1.3x- HBVΔX virions (both genotype D) at 500–1000 viral genome equivalents per cell in media containing 4% PEG 8000 for 16 hours at 37°C. Mock-infected cells were treated with media containing 4% PEG 8000 in the absence of virus. At the end of the infection period, cells were washed three times with William’s E Medium (ThermoFisher Scientific) and cultured in maintenance medium containing 2% DMSO and 2% FBS. The PHH medium was subsequently changed every 2–4 days until the end of the study. For the RNA interference studies, PHH were transfected with 10 nM siRNA using Lipofectamine RNAiMax according to the manufacturer's instructions (Life Technologies) and were infected 3–6 days later.

### Human liver chimeric uPA-SCID mice

Seven male uPA-SCID mice were transplanted with 1x10^6^ PHH from a single healthy donor as described previously [13]. After 10–13 weeks, five of these mice were infected by intraperitoneal injection with 5x10^5^ genome equivalents of cell culture derived HBV genotype C. These animals were sacrificed and serum and liver specimens were collected for measurement of HBV infection 14 weeks later. Serum HBV DNA reached a titer of >1.5x10^7^ copies/mL and serum HBsAg levels were ≥3.2x10^2^ IU/mL in all infected animals. As a control, two of the seven mice were left uninfected. These animals were sacrificed at 15 weeks post-transplantation and then processed identically to the HBV-infected animals.

### HBeAg, HBsAg and cytokine measurements

Hepatitis B e antigen (HBeAg) and Hepatitis B surface antigen (HBsAg) were detected in culture media at the indicated time by ELISA or electrochemiluminescence assay (MSD). The HBeAg and HBsAg ELISAs were performed using the HBeAg EIA kit (International Immuno-Diagnostics, Foster City, CA) and HBsAg ETI-MAK-2 plus kit (DiaSorin, Stillwater, MN), respectively. Concentrations were calculated by interpolation from standard curves with purified HBeAg and HBsAg. The MSD assay was performed according to the manufacturer's instructions (Meso Scale Diagnostics, Rockville, MD). Briefly, cultured supernatants were inactivated with 0.5% Triton X-100 (30 minutes at 37°C) and then transferred into plates pre-spotted with both an anti-HBeAg antibody (Genway Bio, San Diego, CA) and a custom anti-HBsAg antibody. The plates were then incubated for 2 hours at room temperature with gentle shaking, followed by a wash step in PBS with 0.5% Tween. MSD Sulfate tags anti-A and anti-B (1 μg/mL each) were then added to the wells and the plates incubated for a further 2 hours at room temperature with gentle shaking, followed by another wash step in PBS with 0.5% Tween. A 2X solution of MSD T Buffer Read was then added and the plate was read on a Sector Imager 6000 plate scanner. Cytokine levels (n = 36) were measured by luminex assay (Affymetrix, Santa Clara, CA) on a Luminex 200™ instrument accordingly to the manufacturer's instructions. When HBeAg, HBsAg or cytokine levels were below the lower limit of quantitation (LLOQ), the LLOQ value was plotted. If analyte levels were greater than the upper limit of quantitation (ULOQ), the value extrapolated from the standard curve was plotted.

### Epifluorescence microscopy

PHH were fixed in 4% paraformaldehyde and permeabilized with 0.3% Triton-X100 for 30 minutes at room temperature. Cells were stained with rabbit polyclonal anti-HBV core antibody (Dako, Carpinteria, CA) diluted 1:1600 in PBS containing 0.3% Triton-X100 and 3% bovine serum albumin (BSA). After washing with PBS, bound antibodies were detected using Alexa Fluor®-555-conjugated donkey anti-rabbit secondary antibody (Life Technologies) diluted 1:1000 in PBS containing 0.3% Triton-X100 and 3% BSA. Nuclei were stained with 4’, 6-diamidine-2-phenylindole (DAPI) (Life Technologies). Images were taken with an Axiovert 200 microscope (Zeiss, Thornwood, NY). Immunofluorescence analysis of humanized mouse liver tissues was performed as previously described [13]. Tissue sections were stained with 1:200 mouse monoclonal anti-human Smc6 (Abgent, San Diego, CA) and 1:200 rabbit polyclonal anti-PML (Abcam, Cambridge, UK) antibodies diluted in a 1:1 solution of Rodent Block M (BioCare Medical) and Renoir Red diluent (BioCare Medical). Alexa Fluor 488 donkey anti-mouse and Alexa Fluor 594 donkey anti-rabbit were used as secondary antibodies at a 1:1000 dilution in PBS. Images were acquired using a 40X objective lens and an inverted epifluorescence microscope (Leica DM LB, Wetzlar, Germany) and arranged using Adobe Photoshop CS6. A detailed description of all antibodies used is provided in S6 Table.

### Confocal microscopy

Confocal analysis of PHH was performed as previously described [13]. Human Smc6 and Smc5 were detected using primary monoclonal mouse anti-human Smc6 (Abgent) diluted 1:500 and polyclonal rabbit anti-Smc5 (Bethyl Laboratories, Montgomery, TX) diluted 1:100. HBV antigens were detected using primary antibodies polyclonal rabbit anti-HBV core (Dako) diluted 1:1600 and polyclonal rabbit anti-HBsAg (Virostat, Westbrook, ME) diluted 1:500. ND10 core organizing proteins were detected using primary monoclonal mouse anti-PML (Santa Cruz, Santa Cruz, CA) diluted 1:100, polyclonal rabbit anti-PML (Abcam) diluted 1:100 and polyclonal rabbit anti-Sp100 (Novus Biologicals, Littleton, CO) diluted 1:100. Secondary antibodies Alexa Fluor 488 goat anti-mouse, Alexa Fluor 488 goat anti-rabbit, Alexa Fluor 594 goat anti-mouse and Alexa Fluor 594 goat-anti-rabbit (all Life Technologies) were each diluted 1:200 in 3% normal goat serum. Images were acquired using an upright Zeiss LSM880 or LSM710 confocal system equipped with a 63X objective lens (NA 1.4) and Zen software. All images within each sample set were captured using identical confocal settings. Nuclear mean fluorescence intensity values and colocalization measurements between red and green channels were collected using Imaris 8.2.1 image analysis software (BitPlane, Belfast, UK). PML, Sp100, Smc5 and Smc6 nuclear foci (diameter ≥ 0.5 μm) were also counted using Imaris. Images were adjusted equally within each data set for brightness and contrast using Adobe Photoshop CS6. A detailed description of all antibodies used is provided in S6 Table.

### Western blotting

Western blot analysis of PHH was performed as previously described [13]. Membranes were probed with 1:1000 monoclonal mouse anti-human Smc6 (Abgent), 1:1000 polyclonal rabbit anti-PML (Novus Biologicals), 1:1000 monoclonal mouse anti-Sp100 (Abcam), and 1:1000 mouse monoclonal anti-GAPDH (Novus Biologicals). IRDye 680RD goat anti-rabbit or IRDye 800CW goat anti-mouse IgG (Licor, Lincoln, NE) at a 1:5000 dilution were used as secondary antibodies. Blots were visualized using an Odyssey Infrared Imaging System (Licor). Smc6, PML and Sp100 protein levels were quantified by densitometry with the Odyssey software (LiCor) and expressed relative to GAPDH. A detailed description of all antibodies used is provided in S6 Table.

### Northern blotting

Total cell RNA was purified by Trizol (ThermoFisher Scientific). RNA was mixed in a 1:1 v/v ratio with glyoxal sample buffer (Lonza, Basel, Switzerland) and heated at 65°C for 15 minutes followed by 2 minutes on ice. Gel loading buffer was added (Teknova, Hollister, CA) and the sample loaded onto a 1% agarose gel (Agarose MP, Roche, Basel, Switzerland) made with 1X MOPS (Lonza), and DEPC-treated water (ThermoFisher Scientific). RNA markers (Millennium RNA Markers, ThermoFisher Scientific) were included for size determination and treated the same as experimental samples as described above. RNA was electrophoresed at 100V for 3 hours. Prior to transfer, the gel was rinsed briefly in 20X SSC (G-Biosciences, St. Louis, MO). RNA was then transferred to a Nytran SuPerCharge membrane using the Whatman TurboBlotter kit according the manufacturer’s instructions (GE Healthcare, Amersham, United Kingdom). Following transfer, the membrane was washed in 2X SSC (G-Biosciences) for 5 minutes and then cross-linked by exposure to UV light (254 nM) in a CL-1000 Ultraviolet Stratalinker (UVP, Upland, CA) for a total dose of 120 mJ/cm^2^. HBV RNA was detected using a Quantigene 2.0 probe set designed against the HBx region at 55°C overnight according the manufacturer’s instructions (Affymetrix). The chemiluminescent signal was measured with CDP-STAR Detection Reagent (GE Healthcare) and scanned at 425 nm using an ImageQuant LAS4000 (GE Healthcare) and associated software.

### Southern blotting

DNA was isolated from the nuclei of HBV-infected PHH by Hirt extraction as previously described [61]. A total of 1 μg of the extracted DNA was digested with 10 units of T5 exonuclease (New England Biolabs, Ipswich, MA) for 2 hours at 37°C. The digested DNA was then purified using a DNA clean and concentrator kit (Zymo Research, Irvine, CA). Gel loading dye (New England Biolabs) was added and the sample loaded onto a 1.2% TAE agarose gel (Roche, Basel, Switzerland). A 1 Kb DNA ladder (New England Biolabs) was included for size determination. The DNA was electrophoresed at 25V for 21 hours. The gel was then soaked in 0.2 M HCL (Sigma, St. Louis, MO) for 10 minutes, followed by 1 hour incubation with slow shaking in first denaturing buffer and then neutralizing buffer (G-Biosciences). Finally the gel was submerged in 20X SSC (G-Biosciences) for 30 minutes. The DNA was then transferred to a Nytran SuPerCharge membrane using the Whatman TurboBlotter kit, according the manufacturer’s instructions (GE Healthcare). After a 3 hour transfer, the membrane was rinsed in 2X SSC (G-Biosciences) and then cross-linked by exposure to UV light (254 nM) in a CL-1000 Ultraviolet Stratalinker (UVP) for a total dose of 120 mJ/cm^2^. HBV DNA was then detected by branched DNA. Briefly, the membrane was hybridized with Quantigene 2.0 HBV DNA probes in hybridization buffer at 55°C overnight. After washing with 1X washing buffer, the membrane was treated for 1 hour at 55°C with first Quantigene 2.0 Pre-Amplifier and then Amplifier in Amplifier Diluent. The DNA was subsequently labeled with Quantigene 2.0 label probe at 50°C for 1 hour. HBV cccDNA was detected using the CDP-STAR Detection Reagent (GE Healthcare) with an ImageQuant LAS4000 (GE Healthcare). All reagents used for branched DNA hybridization and amplification were obtained from Affymetrix.

### RNA-Seq analysis

Isolation of total cellular RNA and RNA-Seq was conducted by Expression Analysis (Durham, NC) as described previously [62]. On-column DNase I treatment was performed during RNA isolation with the RNeasy Mini Kit (Qiagen). cDNA libraries were constructed using a TruSeq Stranded mRNA Library Prep Kit (Illumina, San Diego, CA). Pair-end sequencing was conducted using Illumina HiSeq2000 with read length of 50 nucleotides. On average, approximately 30 million reads were generated per sample. Sequencing reads were aligned to the human and HBV genomes by STAR [63]. The Bioconductor packages edgeR [64] and limma [65] were used to normalize sequence count data and conduct differential gene expression analysis. False discovery rate (FDR) was calculated using the Benjamini-Hochberg method [66]. All differentially expressed genes (DEGs) had an absolute fold change >2 and FDR <0.05, and passed a low-expression filter. Principal component analysis (PCA) was performed in R (https://www.r-project.org/). Pathway analysis was performed using Ingenuity^®^ Pathway Analysis (IPA; Qiagen).

### Quantitative RT-PCR

Total cellular RNA was isolated from PHH cultured in 96-well plates using an RNeasy 96 Kit (Qiagen) following the manufacturer’s instructions. Real-Time RT-PCR was performed with TaqMan® Fast Virus 1-Step Master Mix (Life Technologies) using a QuantStudio 7 Flex Real-Time PCR System (Life Technologies) following the manufacturer’s instructions. β-actin mRNA expression was used to normalize target gene expression. All oligonucleotide primer sets were manufactured by Life Technologies.

### Flow cytometry

PHH were thawed, washed once with PBS and immediately fixed with 4% paraformaldehyde for 30 minutes on ice. After fixation, the cells were pelleted and blocked for 30 minutes on ice in PBS supplemented with 5% normal goat serum, 5% normal rabbit serum and 0.1% BSA for staining of cell-surface markers, or according to the manufacturer’s instructions for the eBioscience Intracellular Staining Kit (eBioscience, San Diego, CA) when detecting intracellular antigens. Next, cells were incubated with anti-CD45-FITC, anti-CD81-APC or anti-CD68-FITC (all BD Biosciences, San Jose, CA), anti-HLA-DR-FITC or anti-CD299-APC (both eBioscience), anti-human albumin-FITC (Rockland, Limerick, PA) or isotype-matched controls for 60 minutes on ice. All antibodies were used at manufacturer recommended concentrations except for anti-human albumin-FITC, which was used at 10 μg/mL. After staining, PHH were washed three times with PBS before being assessed by flow cytometry using a Fortessa LSR instrument (BD Biosciences). All flow cytometry data was analyzed using FlowJo V10.08r1 software (TreeStar Inc., Ashland, OR). A detailed description of all antibodies used is provided in S6 Table.

### Statistical analysis

Statistical significance was tested using a two-tailed, paired t-test (for two sample comparisons) or one-way ANOVA with Dunnett's multiple comparison correction data (for multiple comparisons). A value of *p*<0.05 was considered significant.

## Supporting Information

S1 Fig Smc5/6 co-localizes with ND10 components in PHH. (a) Representative confocal microscopy images of uninfected PHH stained for Smc5 (red), Smc6 (green), PML (green) or Sp100 (red). Nuclei were stained with DAPI (blue). Scale bars represent 10 μm. Nuclei are outlined by white dotted lines in all images. (b) Quantitation of the confocal data from (a). The number of Smc5, Smc6 or PML nuclear foci containing a second protein (PML, Sp100 or Smc5) is expressed as the percentage of the total Smc5, Smc6 or PML foci detected in each nucleus. The bar height indicates the mean and the errors bars represent the standard error of the mean. The number of nuclei analyzed for each comparison is displayed above the plot.
(TIF)

S2 Fig Levels of Smc6, PML and Sp100 under infection and knock-down conditions. PHH were transfected with siRNA to the indicated gene(s) or were mock-transfected (mock), and incubated for 3 days before infection with (a) wild-type HBV or (b) HBVΔX. Lysates were prepared for Western blot analysis at day 14 post-infection. Representative Western blots from experiments performed with two independent PHH donors are shown on the left. The blots have been cropped for ease of presentation. All gels were run under the same experimental conditions and full-length blots are presented in S16 Fig. Quantitation of all blots (n = 2) is shown on the right; the bar height indicates mean levels expressed as a percentage of mock-transfected cells and the errors bars represent the standard error of the mean. In a separate experiment, it was shown the siCtrl did not affect Smc6, PML or Sp100 protein levels in either HBV or HBVΔX-infected PHH.
(TIF)

S3 Fig Extracellular HBeAg correlates with intracellular HBV mRNA levels. PHH were seeded at day -4, transfected with the indicated siRNA on day -3 and infected on day 0 with HBV or HBVΔX. HBV and GAPDH RNA (top) and HBeAg (bottom) were analyzed at 13 days post-infection. The Northern blots have been cropped for ease of presentation. All gels were run under the same experimental conditions and full-length blots are presented in S16 Fig. The HBeAg data is plotted directly below the corresponding Northern Blot lane. As previously described [13], knock-down of DDB1 prevents HBx targeting Smc5/6 for degradation and leads to transcriptional silencing of HBV cccDNA. Conversely, knock-down of Smc6 rescues cccDNA transcription in the absence of HBx.
(TIF)

S4 Fig HBx RNA is detected early after HBV infection of PHH. RNA-Seq reads at various time-points after HBV-infection or mock-infection (-) of PHH (donor 2). The study design is outlined in Fig 6A. The x-axis denotes the HBV genome position by reference to the schematic above and the y-axis displays the sequencing coverage of the HBV genome, with the range in parentheses (note the different scale for different time-points). The time-point post-infection is shown adjacent to the y-axis. The green box denotes the HBx transcript region using the canonical transcription start site. h: hour, d: day, bp: base-pair.
(TIF)

S5 Fig HBx RNA is detected early after HBVΔX infection of PHH. RNA-Seq reads at various time-points after HBVΔX-infection of PHH. Note that the HBVΔX virus produces HBx RNA containing a premature stop at the 7^th^ amino acid position after the first ATG in the HBx open reading frame [7]. The x-axis denotes the HBV genome position by reference to the schematic above and the y-axis displays the sequencing coverage of the HBV genome, with the range in parentheses (note the different scale for different time-points). The time-point post-infection is shown adjacent to the y-axis. The green box denotes the HBx transcript region using the canonical transcription start site. h: hour, bp: base-pair.
(TIF)

S6 Fig The PHH transcriptome changes over time, but is not substantially modified by HBV infection. (a) Schematic summarizing the design of a pilot study to evaluate host response to high level HBV infection in PHH. Biological replicates (n = 10) were set-up at each time-point for each condition and were pooled prior to transcriptome analysis. Cell culture media was changed on days 1, 3, 6, 8 and 10. d: day, h: hour. (b) Immunofluorescence staining of HBV core (red) and nuclei (blue) at various times post-infection with HBV (+) or mock (-). (c) Principle component analysis of the host transcriptome at various times post-infection. Principal components (PC) #1 and #2 are plotted on the x- and y-axis, respectively.
(TIF)

S7 Fig Transcriptional signatures of PHH genes up-regulated post-seeding. Ingenuity pathway analysis of genes up-regulated (n = 913) over time in mock-infected PHH (study outlined in S6 Fig). All genes had a fold change >2 on day 13 post-infection, and a temporal correlation r≥0.8 with p<0.05 (Pearson correlation). Pathway enrichment was calculated with the Fisher’s exact test with multiple testing correction by the Benjamini and Hochberg method. The–log(*p*-value) for *p* = 0.05 and *p* = 0.01 significance levels are indicated.
(TIF)

S8 Fig Transcriptional signatures of PHH genes down-regulated post-seeding. Ingenuity pathway analysis of genes down-regulated (n = 1641) over time in mock-infected PHH (study outlined in S6 Fig). All genes had an absolute fold change >2 on day 13 post-infection, and an absolute temporal correlation r≥0.8 with p<0.05 (Pearson correlation). Pathway enrichment was calculated with the Fisher’s exact test with multiple testing correction by the Benjamini and Hochberg method. The–log(*p*-value) for *p* = 0.05 significance level is indicated.
(TIF)

S9 Fig Expression of select genes in mock-infected PHH over time. RNA-Seq data of mock-infected PHH expressed as fold-change relative to the 4 hour time-point (study outlined in S6 Fig). The Pearson correlation coefficients for correlation with time are indicated on each plot. All genes passed a low expression filter.
(TIF)

S10 Fig Infection of PHH at different times post-seeding. PHH were infected at 1, 2, 6 or 9 days post-seeding with HBV (+) or mock-infected (-), and stained for HBV core (red) at 13 days (d) post-infection. Nuclei were stained with DAPI (blue).
(TIF)

S11 Fig Innate immune stimuli induce ISG expression in PHH. qRT-PCR data of mock-infected PHH from two independent donors treated with 100 IU/mL IFN-α, 10 μg/mL poly(I:C) or 10 HAU/mL Sendai virus (SeV) for 8 hours. The bar height indicates the mean fold-change relative to the no treatment (Tx) control and the errors bars represent the standard error of the mean. PHH donors were from the studies described in Fig 6 and S6 Fig. Note that *IFI44L* and *ZBP1* (DAI) were amongst the most highly induced ISGs in the RNA-Seq analysis of PHH treated with IFN-α and poly(I:C) (Fig 7).
(TIF)

S12 Fig High level infection of PHH with HBVΔX. PHH were seeded at day -4, mock-transfected (no siRNA) or transfected with the indicated siRNA on day -3 and infected on day 0 with HBVΔX or HBV. HBV core (red) was analyzed at 13 days post-infection. Nuclei were stained with DAPI (blue). Mean HBeAg levels in HBV-infected PHH were comparable (339–363 ng/mL), irrespective of the siRNA treatment. In contrast, HBeAg were produced by siSmc6-treated HBVΔX-infected PHH (mean = 2161 ng/mL), but was not detected with mock- or siCtrl-transfected HBVΔX-infected PHH.
(TIF)

S13 Fig HBVΔX does not induce ISG expression in PHH. PHH were seeded at day -4, mock-transfected (none) or transfected with the indicated siRNA on day -3 and infected on day 0 with wild-type HBV or HBVΔX. HBV RNA, HBeAg and ISG mRNAs were analyzed at day 13 post-infection. The HBV RNA and HBeAg data is expressed as a percentage of no siRNA (wild-type HBV) or siSmc6-treated cells (HBVΔX). Mean HBeAg levels in mock-transfected PHH infected with wild-type HBV or siSmc6-treated PHH infected with HBVΔX were 431 and 1946 ng/mL, respectively. Since PHH infected with wild-type HBV do not induce an ISG response (Fig 7, S2 Table), ISG mRNA data for both wild-type HBV and HBVΔX were expressed as a percentage of mock-transfected PHH infected with wild-type HBV. In all plots, the bar height indicates the mean and the errors bars represent the standard error of the mean of n = 3 independent experiments.
(TIF)

S14 Fig Model proposing the role of Smc5/6 complex and ND10 in the HBV replication cycle. We hypothesize that ND10 localizes to cccDNA in HBV-infected hepatocytes as it does with other DNA viral genomes. HBx redirects the DDB1 E3 ubiquitin ligase and promotes the degradation of Smc5/6 associated with ND10, but not PML or Sp100. It is not known whether HBV infection otherwise alters the protein composition of ND10. It is also not known whether HBV cccDNA remains associated with ND10 after Smc5/6 degradation. However, our data suggests HBV transcription does not require PML or Sp100.
(TIF)

S15 Fig Purity of PHH assessed by flow cytometry. (a) and (b) are plots for two independent PHH donors. Dashed line depicts control staining. No positive staining of CD68 or CD299 was detected in either donor. Of note, these analyses were performed on freshly thawed PHH cultures and likely over-estimate the frequency of NPCs after several days in culture in 2% DMSO (see S9 Fig; compare temporal changes in CD68 (Kupffer cell marker) and albumin (hepatocyte marker) mRNA levels).
(TIF)

S16 Fig Full-length Western, Southern and Northern Blots. (PDF)

S1 Table DEGs associated with HBV-infection of PHH. All genes listed were classified as a DEG at one or more time-points in HBV-infected PHH. Fold-change (FC) values are log_2_. FDR values of less than 0.05 are highlighted in pink. The gene list is sorted by FDR at Day 13 in HBV-infected PHH.
(XLSX)

S2 Table HBV infection does not induce ISG expression in PHH. Expression of top IFN-induced genes in PHH infected with HBV or treated with either IFN-α or poly(I:C) expressed as mean log_2_ fold-change (FC) of two independent PHH donors. All genes (n = 100) had a mean fold-change >2 and FDR<0.05 at 8 hours post-treatment with IFN-α relative to the time-matched mock-infected control, and passed a low-expression filter. FDR values of less than 0.05 are highlighted in pink.
(XLSX)

S3 Table HBV infection does not induce cytokines in PHH (donor 1). Cytokines from PHH infected with HBV or mock-infected, or treated with either IFN-α or poly(I:C). ^a^Maximum cytokines levels at any time-point between 4h to 13d post-infection. ^b^Cytokine levels in mock-infected PHH were from time-matched samples. ^c^Maximum cytokines levels in uninfected PHH at any time-point between 4h to 24h post-treatment. LLOQ; lower limit of quantitation. ULOQ; upper limit of quantitation.
(DOC)

S4 Table HBV infection does not induce cytokines in PHH (donor 2). Cytokines from PHH infected with HBV or mock-infected, or treated with either IFN-α or poly(I:C). ^a^Maximum cytokines levels at any time-point between 4h to 13d post-infection. ^b^Cytokine levels in mock-infected PHH were from time-matched samples. ^c^Maximum cytokines levels in uninfected PHH at any time-point between 4h to 24h post-treatment. LLOQ; lower limit of quantitation. ULOQ; upper limit of quantitation.
(DOC)

S5 Table HBVΔX infection does not induce cytokines in PHH. ^a^Maximum cytokines levels at any time-point between 4h to 13d post-infection. ^b^Cytokine levels in mock-infected PHH were from time-matched samples. ^c^Maximum cytokines levels at any time-point between 4h to 24h post-treatment. LLOQ; lower limit of quantitation. ULOQ; upper limit of quantitation.
(DOC)

S6 Table Antibody summary. In addition to the manufacturer's validation, the specificity of the primary antibodies for microscopy and Western blot applications was confirmed by siRNA and/or over-expression analysis. CM: confocal microscopy, Epi: epifluorescence microscopy, WB: Western blot, FC: flow cytometry, n/a: not applicable.
(DOC)
