# Supplementary figures and images for: The Smc5/6 Complex Restricts HBV when Localized to ND10 without Inducing an Innate Immune Response and Is Counteracted by the HBV X Protein Shortly after Infection
Source: PLoS One. 2017 Jan 17;12(1):e0169648. doi: 10.1371/journal.pone.0169648 (PMC5240991; doi:10.1371/journal.pone.0169648)

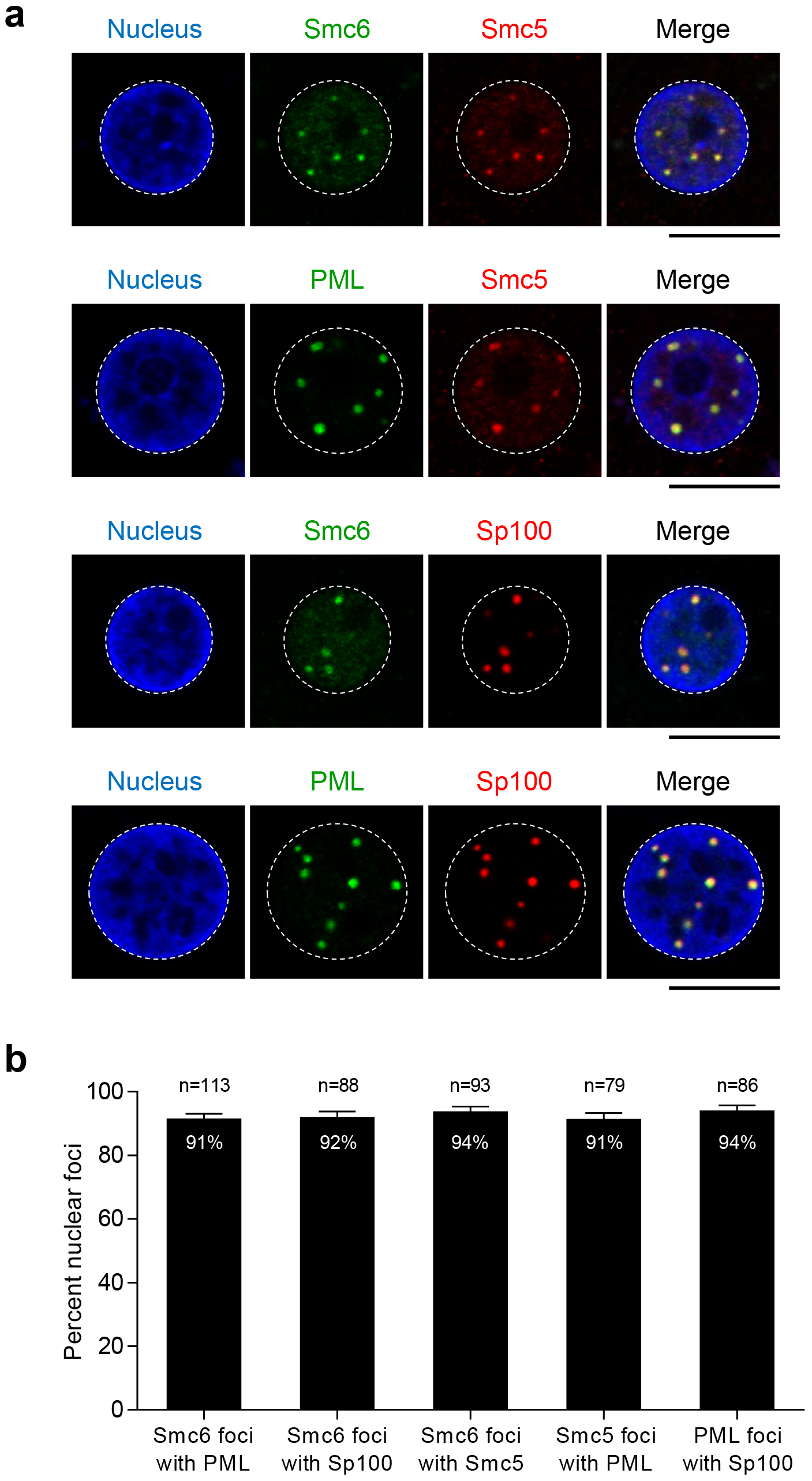

Supplement: S1 Fig — (a) Representative confocal microscopy images of uninfected PHH stained for Smc5 (red), Smc6 (green), PML (green) or Sp100 (red). Nuclei were stained with DAPI (blue). Scale bars represent 10 μm. Nuclei are outlined by white dotted lines in all images. (b) Quantitation of the confocal data from (a). The number of Smc5, Smc6 or PML nuclear foci containing a second protein (PML, Sp100 or Smc5) is expressed as the percentage of the total Smc5, Smc6 or PML foci detected in each nucleus. The bar height indicates the mean and the errors bars represent the standard error of the mean. The number of nuclei analyzed for each comparison is displayed above the plot. (TIF) [file pone.0169648.s001.tif]

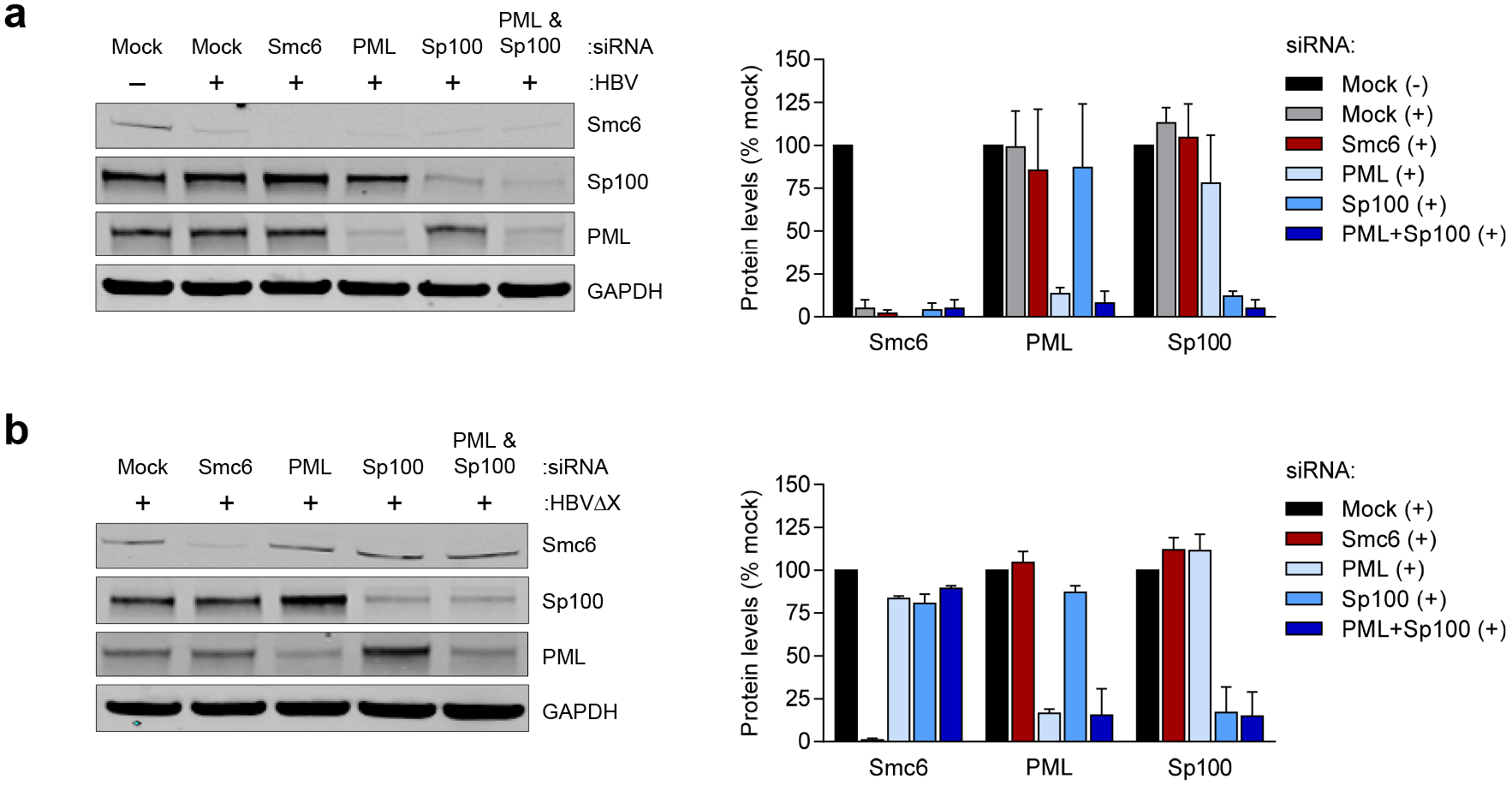

Supplement: S2 Fig — PHH were transfected with siRNA to the indicated gene(s) or were mock-transfected (mock), and incubated for 3 days before infection with (a) wild-type HBV or (b) HBVΔX. Lysates were prepared for Western blot analysis at day 14 post-infection. Representative Western blots from experiments performed with two independent PHH donors are shown on the left. The blots have been cropped for ease of presentation. All gels were run under the same experimental conditions and full-length blots are presented in S16 Fig. Quantitation of all blots (n = 2) is shown on the right; the bar height indicates mean levels expressed as a percentage of mock-transfected cells and the errors bars represent the standard error of the mean. In a separate experiment, it was shown the siCtrl did not affect Smc6, PML or Sp100 protein levels in either HBV or HBVΔX-infected PHH. (TIF) [file pone.0169648.s002.tif]

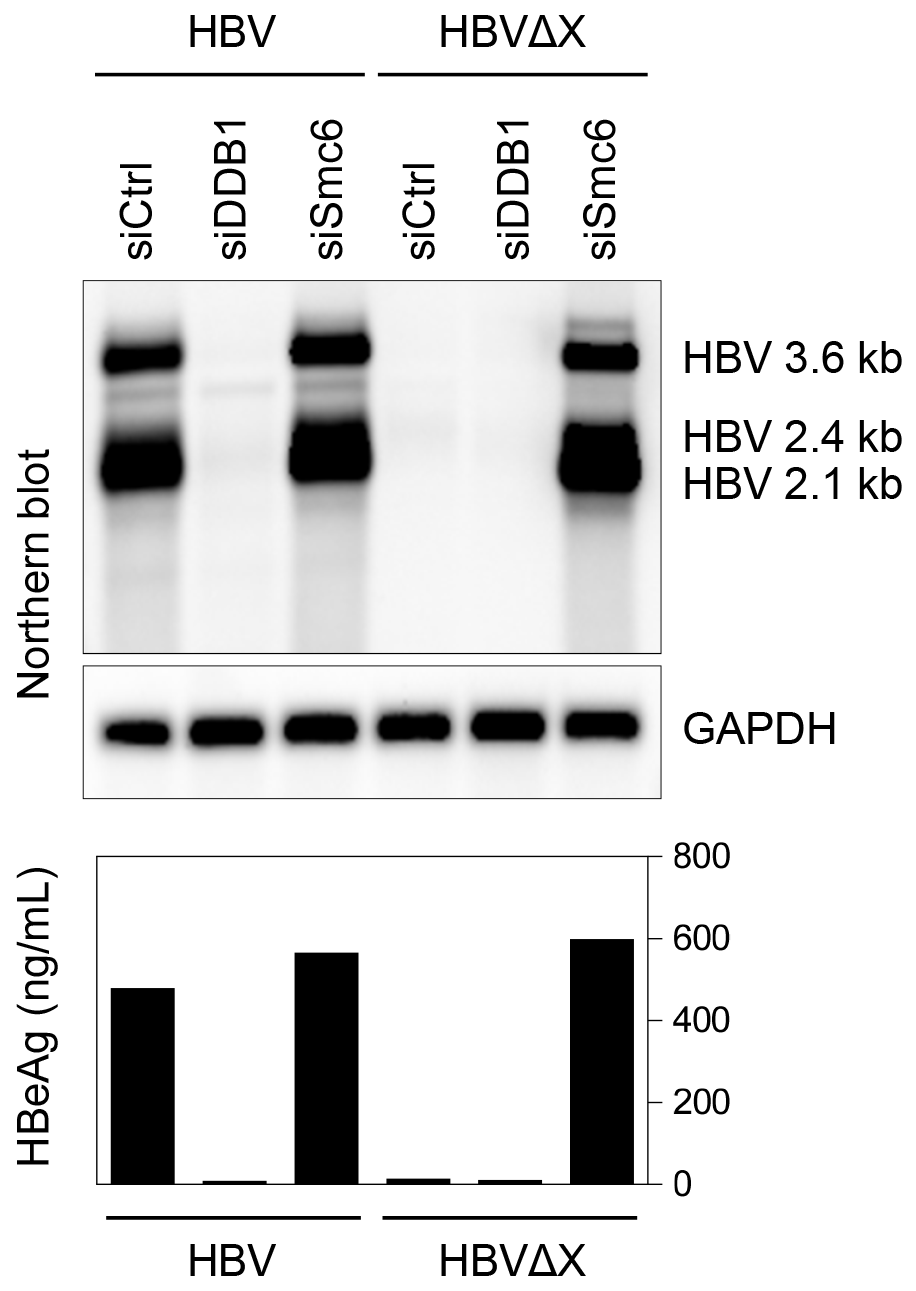

Supplement: S3 Fig — PHH were seeded at day -4, transfected with the indicated siRNA on day -3 and infected on day 0 with HBV or HBVΔX. HBV and GAPDH RNA (top) and HBeAg (bottom) were analyzed at 13 days post-infection. The Northern blots have been cropped for ease of presentation. All gels were run under the same experimental conditions and full-length blots are presented in S16 Fig. The HBeAg data is plotted directly below the corresponding Northern Blot lane. As previously described [13], knock-down of DDB1 prevents HBx targeting Smc5/6 for degradation and leads to transcriptional silencing of HBV cccDNA. Conversely, knock-down of Smc6 rescues cccDNA transcription in the absence of HBx. (TIF) [file pone.0169648.s003.tif]

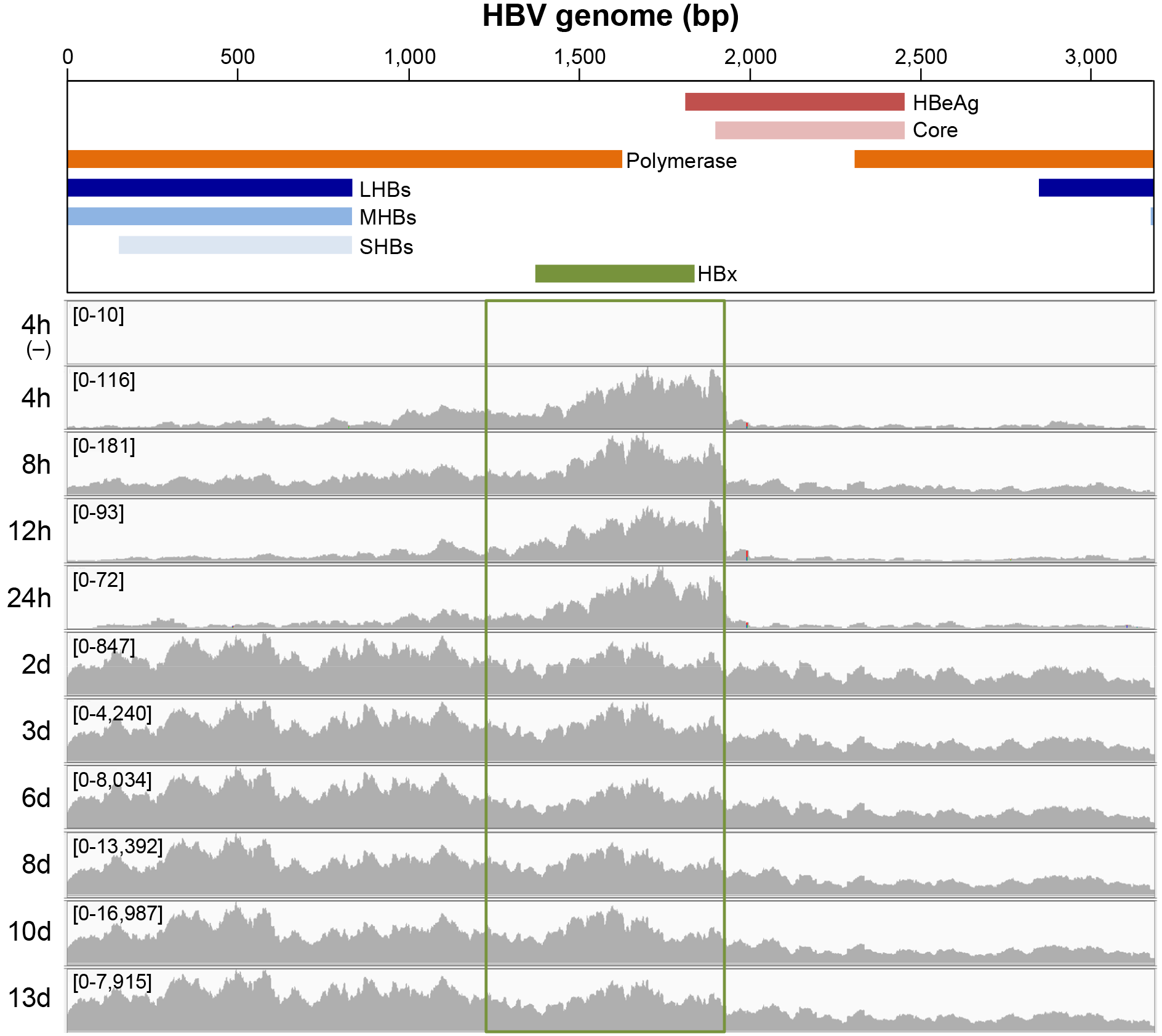

Supplement: S4 Fig — RNA-Seq reads at various time-points after HBV-infection or mock-infection (-) of PHH (donor 2). The study design is outlined in Fig 6A. The x-axis denotes the HBV genome position by reference to the schematic above and the y-axis displays the sequencing coverage of the HBV genome, with the range in parentheses (note the different scale for different time-points). The time-point post-infection is shown adjacent to the y-axis. The green box denotes the HBx transcript region using the canonical transcription start site. h: hour, d: day, bp: base-pair. (TIF) [file pone.0169648.s004.tif]

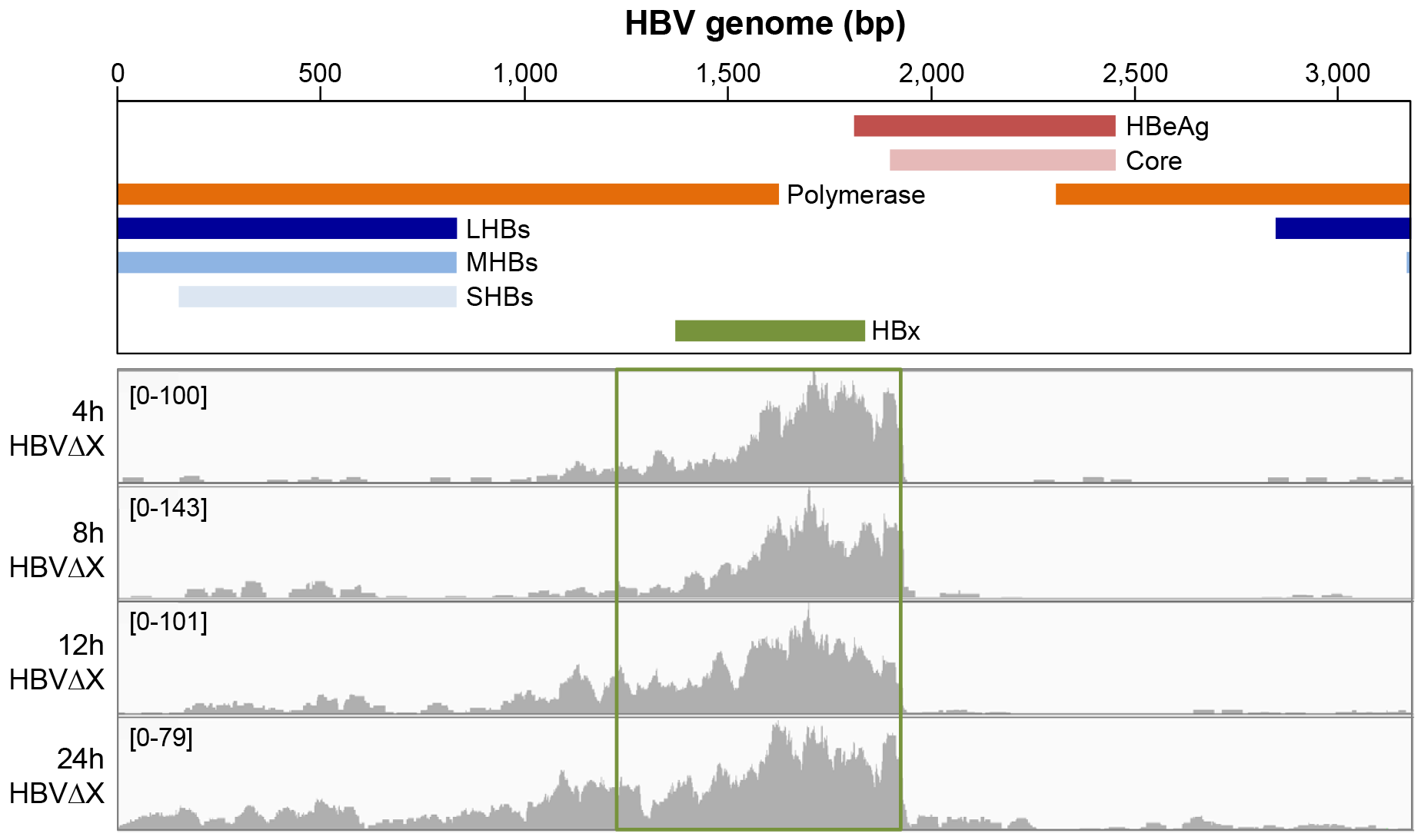

Supplement: S5 Fig — RNA-Seq reads at various time-points after HBVΔX-infection of PHH. Note that the HBVΔX virus produces HBx RNA containing a premature stop at the 7th amino acid position after the first ATG in the HBx open reading frame [7]. The x-axis denotes the HBV genome position by reference to the schematic above and the y-axis displays the sequencing coverage of the HBV genome, with the range in parentheses (note the different scale for different time-points). The time-point post-infection is shown adjacent to the y-axis. The green box denotes the HBx transcript region using the canonical transcription start site. h: hour, bp: base-pair. (TIF) [file pone.0169648.s005.tif]

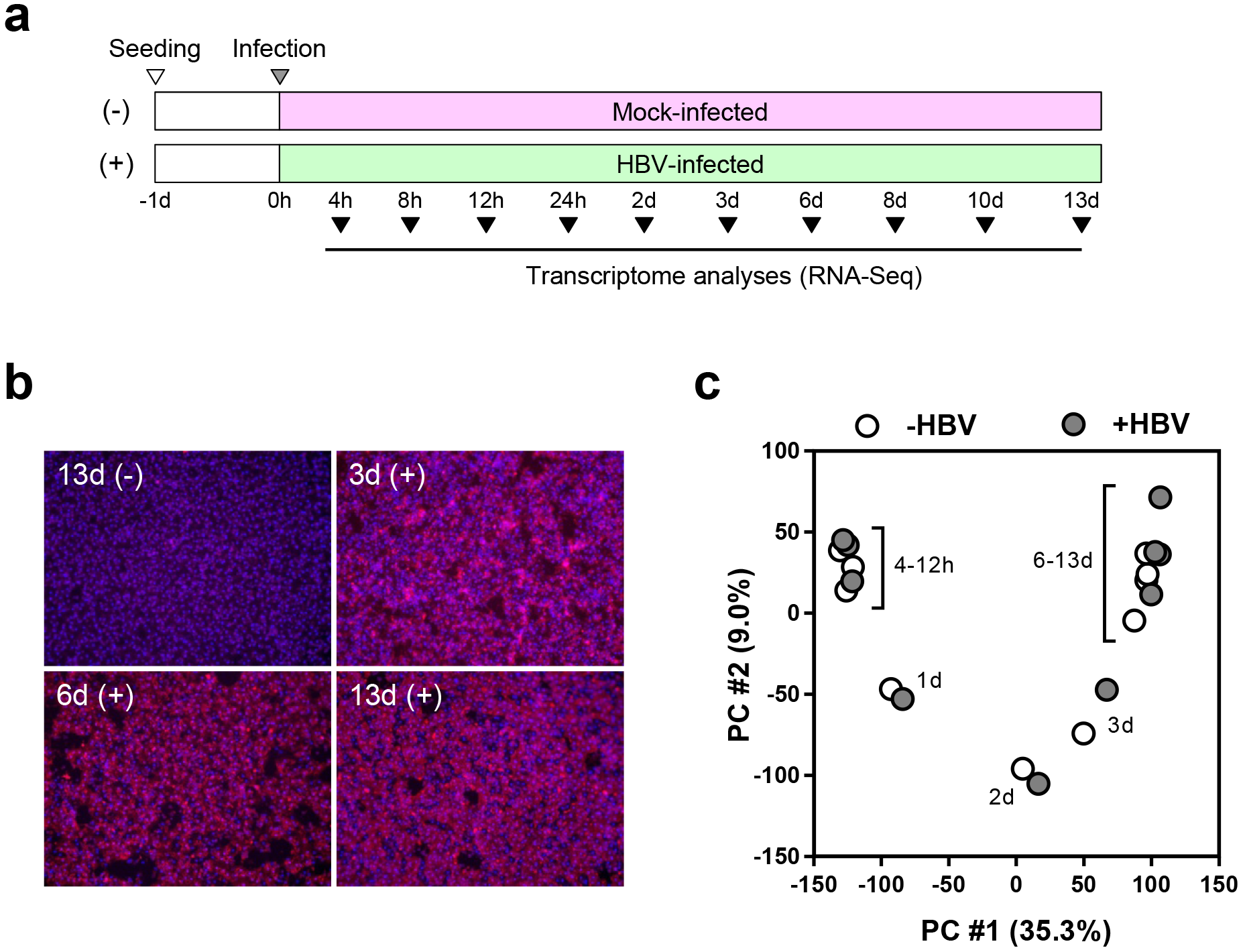

Supplement: S6 Fig — (a) Schematic summarizing the design of a pilot study to evaluate host response to high level HBV infection in PHH. Biological replicates (n = 10) were set-up at each time-point for each condition and were pooled prior to transcriptome analysis. Cell culture media was changed on days 1, 3, 6, 8 and 10. d: day, h: hour. (b) Immunofluorescence staining of HBV core (red) and nuclei (blue) at various times post-infection with HBV (+) or mock (-). (c) Principle component analysis of the host transcriptome at various times post-infection. Principal components (PC) #1 and #2 are plotted on the x- and y-axis, respectively. (TIF) [file pone.0169648.s006.tif]

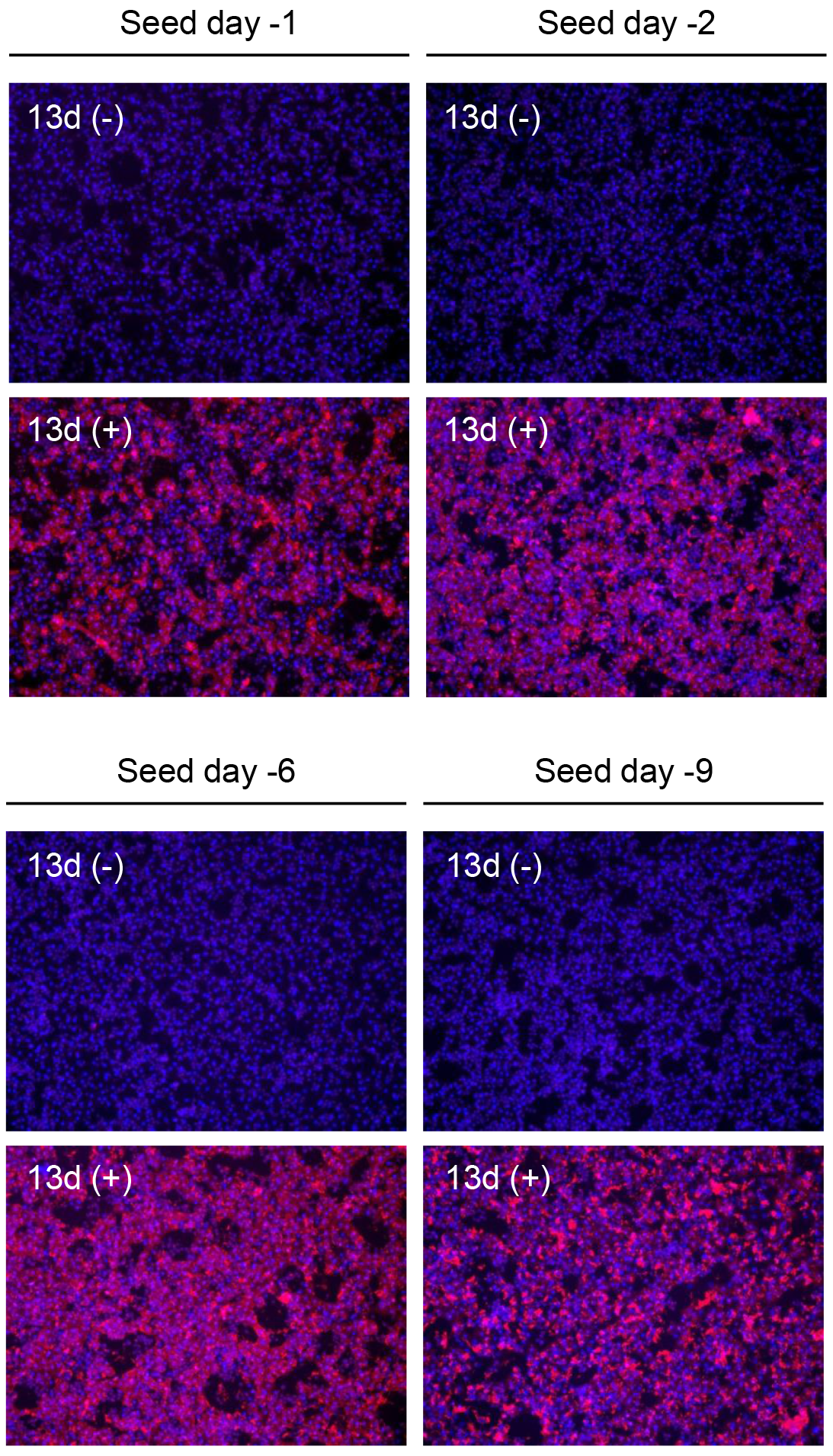

Supplement: S10 Fig — PHH were infected at 1, 2, 6 or 9 days post-seeding with HBV (+) or mock-infected (-), and stained for HBV core (red) at 13 days (d) post-infection. Nuclei were stained with DAPI (blue). (TIF) [file pone.0169648.s010.tif]

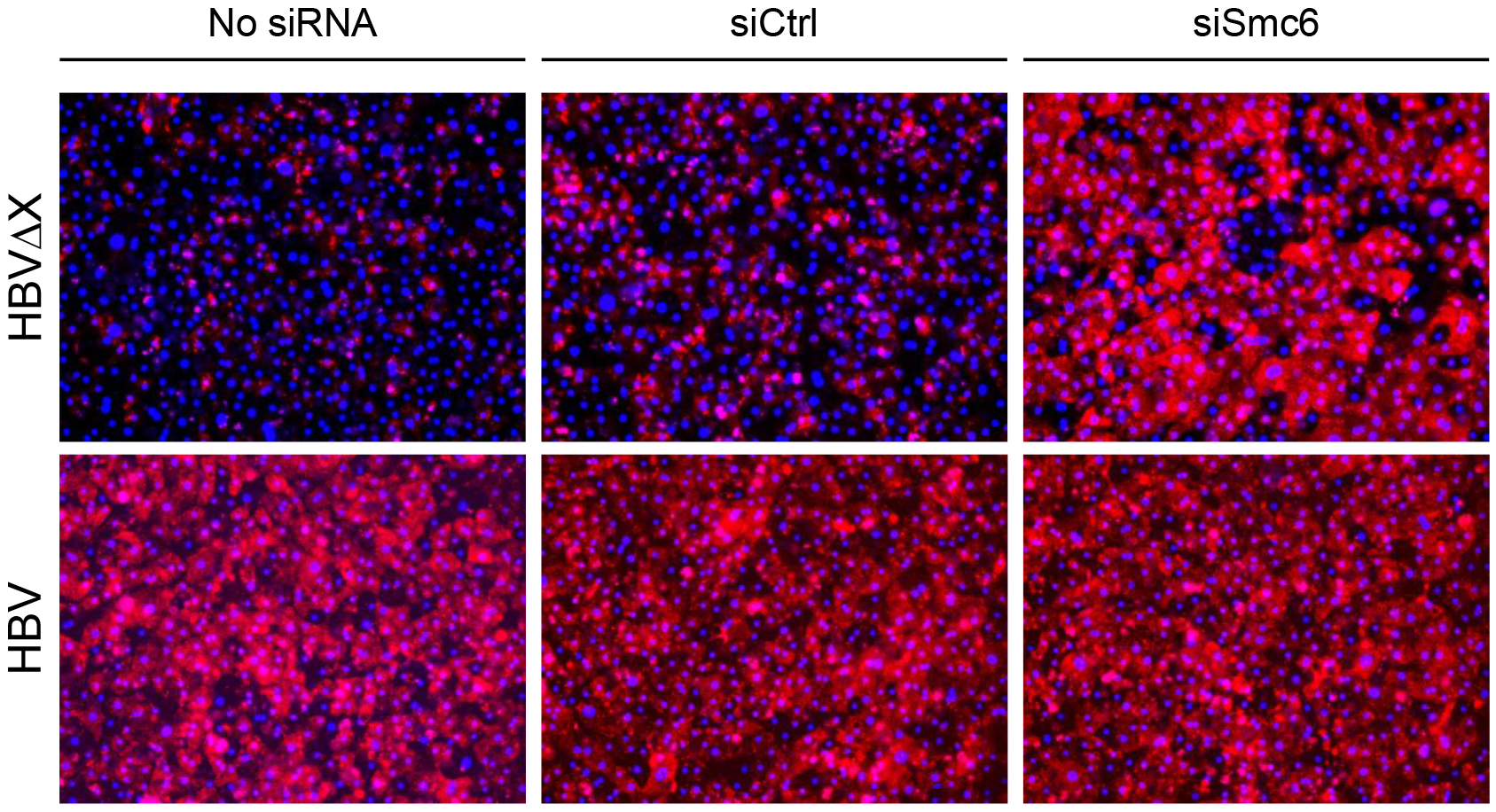

Supplement: S12 Fig — PHH were seeded at day -4, mock-transfected (no siRNA) or transfected with the indicated siRNA on day -3 and infected on day 0 with HBVΔX or HBV. HBV core (red) was analyzed at 13 days post-infection. Nuclei were stained with DAPI (blue). Mean HBeAg levels in HBV-infected PHH were comparable (339–363 ng/mL), irrespective of the siRNA treatment. In contrast, HBeAg were produced by siSmc6-treated HBVΔX-infected PHH (mean = 2161 ng/mL), but was not detected with mock- or siCtrl-transfected HBVΔX-infected PHH. (TIF) [file pone.0169648.s012.tif]

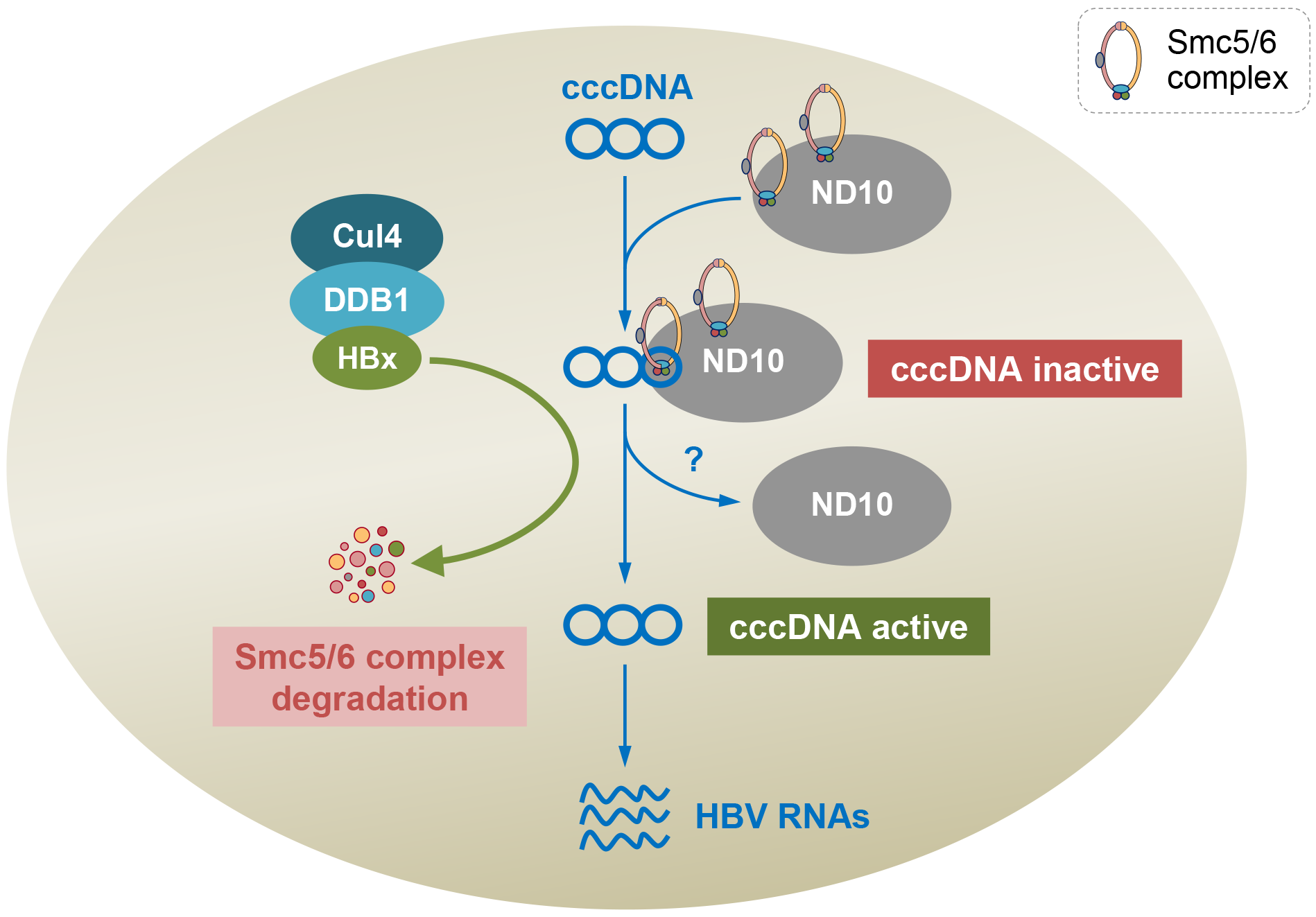

Supplement: S14 Fig — We hypothesize that ND10 localizes to cccDNA in HBV-infected hepatocytes as it does with other DNA viral genomes. HBx redirects the DDB1 E3 ubiquitin ligase and promotes the degradation of Smc5/6 associated with ND10, but not PML or Sp100. It is not known whether HBV infection otherwise alters the protein composition of ND10. It is also not known whether HBV cccDNA remains associated with ND10 after Smc5/6 degradation. However, our data suggests HBV transcription does not require PML or Sp100. (TIF) [file pone.0169648.s014.tif]

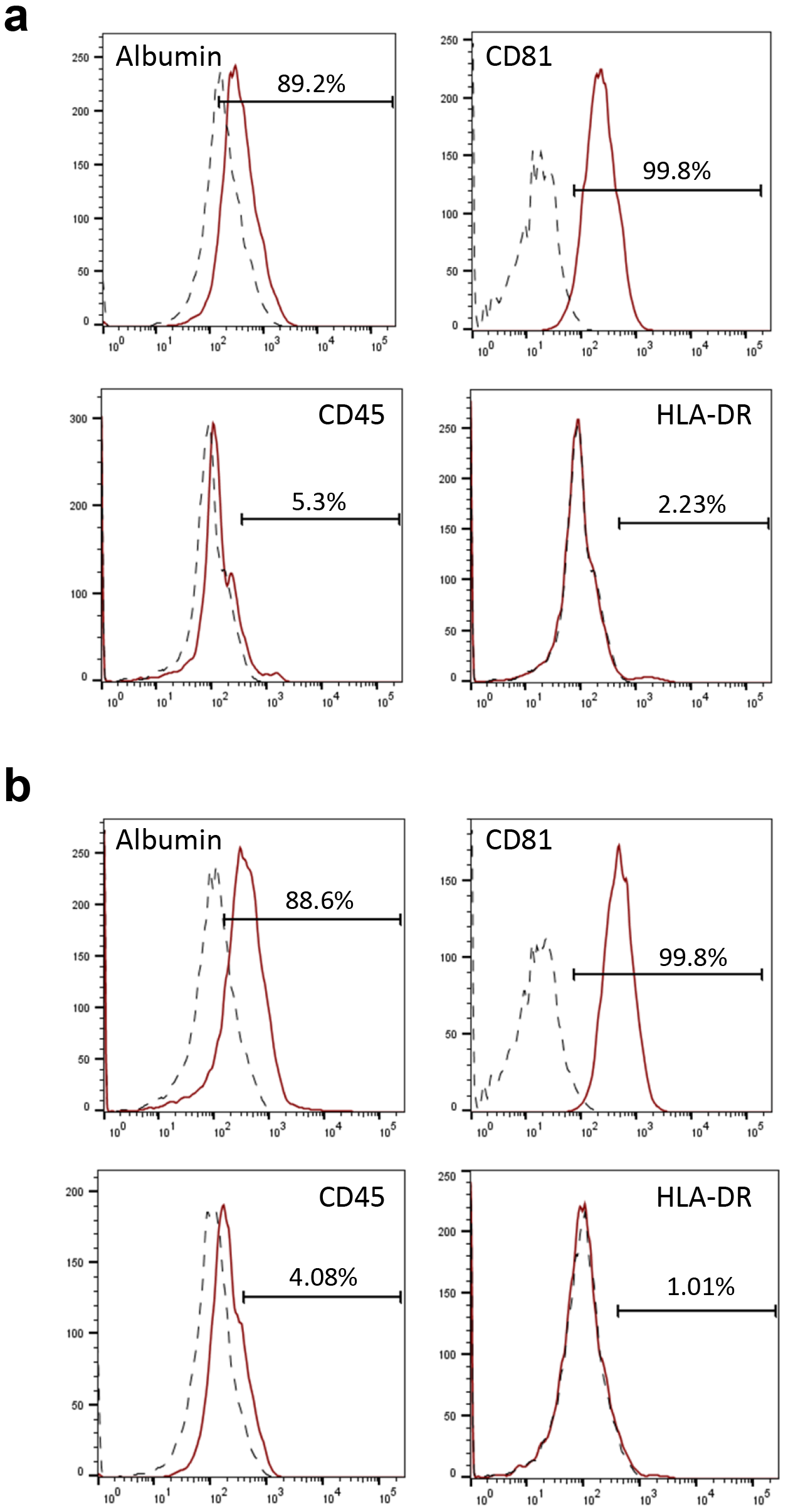

Supplement: S15 Fig — (a) and (b) are plots for two independent PHH donors. Dashed line depicts control staining. No positive staining of CD68 or CD299 was detected in either donor. Of note, these analyses were performed on freshly thawed PHH cultures and likely over-estimate the frequency of NPCs after several days in culture in 2% DMSO (see S9 Fig; compare temporal changes in CD68 (Kupffer cell marker) and albumin (hepatocyte marker) mRNA levels). (TIF) [file pone.0169648.s015.tif]
