# Supplementary material for: The Smc5/6 Complex Restricts HBV when Localized to ND10 without Inducing an Innate Immune Response and Is Counteracted by the HBV X Protein Shortly after Infection
Source: PLoS One. 2017 Jan 17;12(1):e0169648. doi: 10.1371/journal.pone.0169648 (PMC5240991; doi:10.1371/journal.pone.0169648)

Fig. 2a. Western Blots

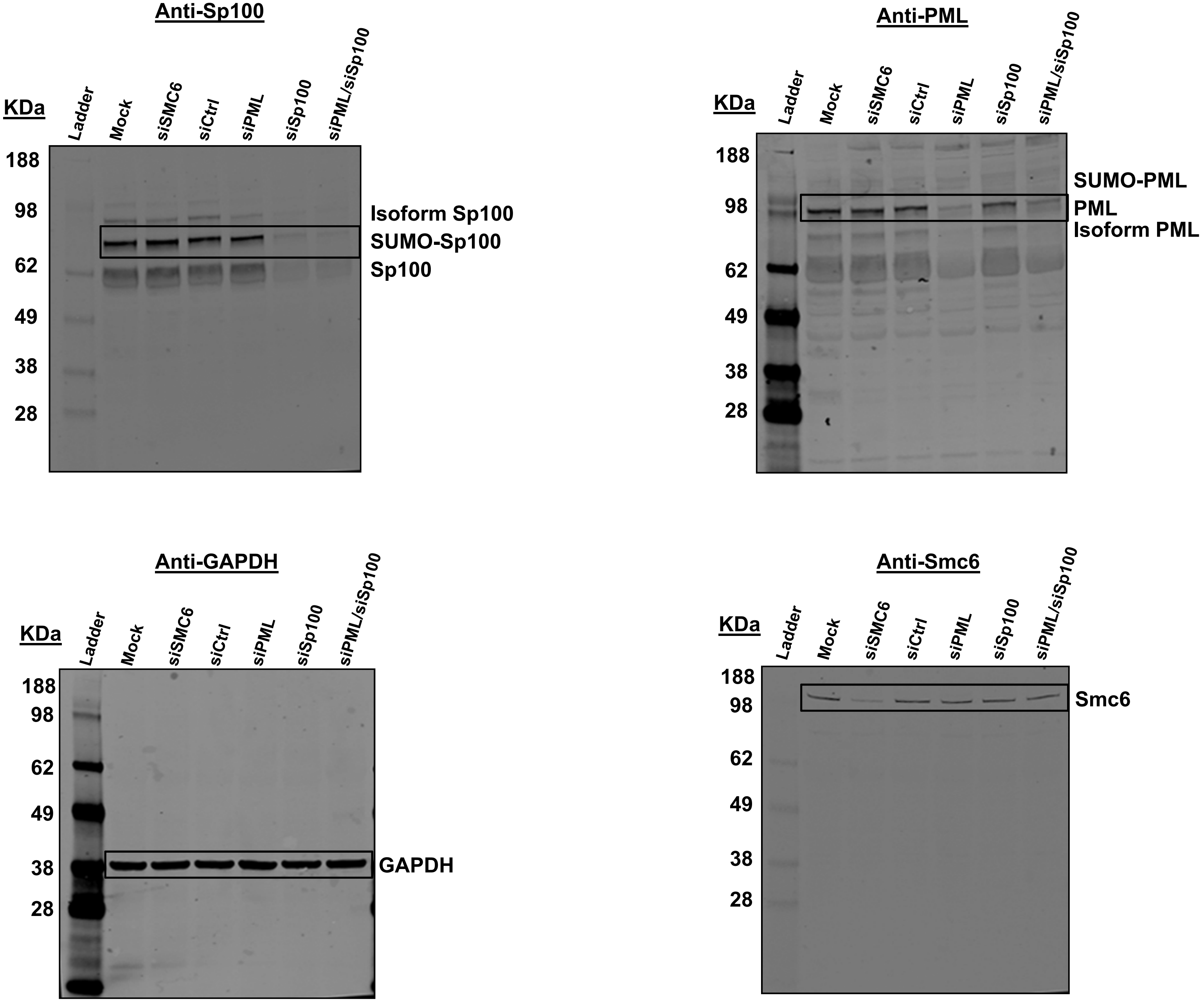

Fig. 3a. Southern blot

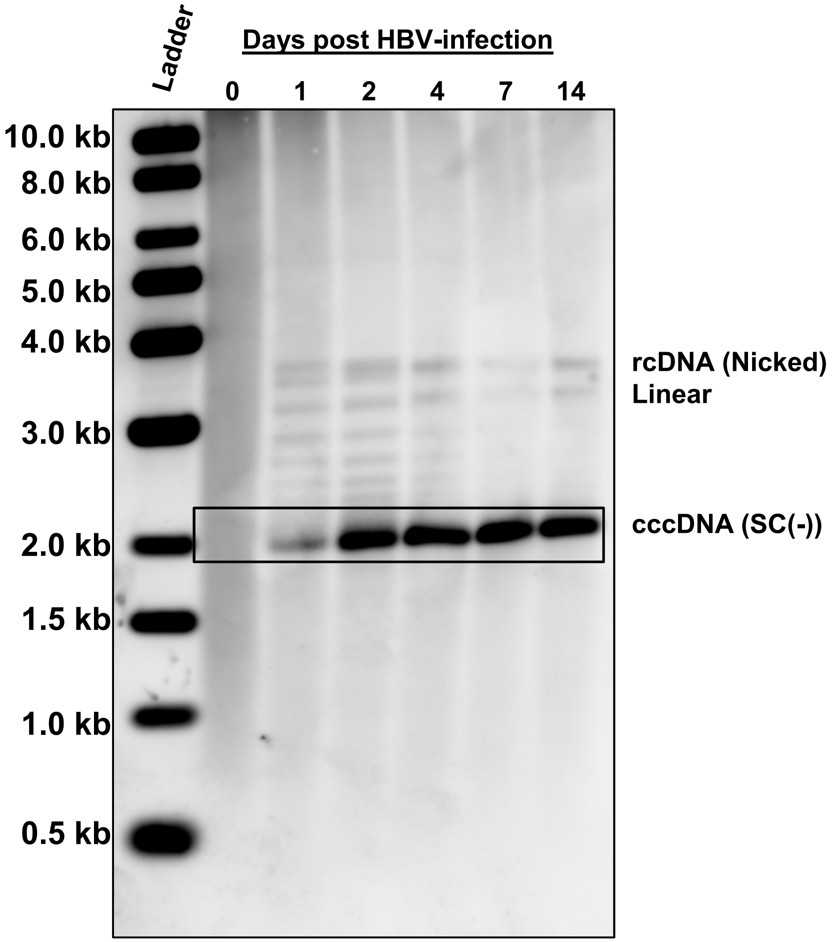

Fig. 3b. Northern blot

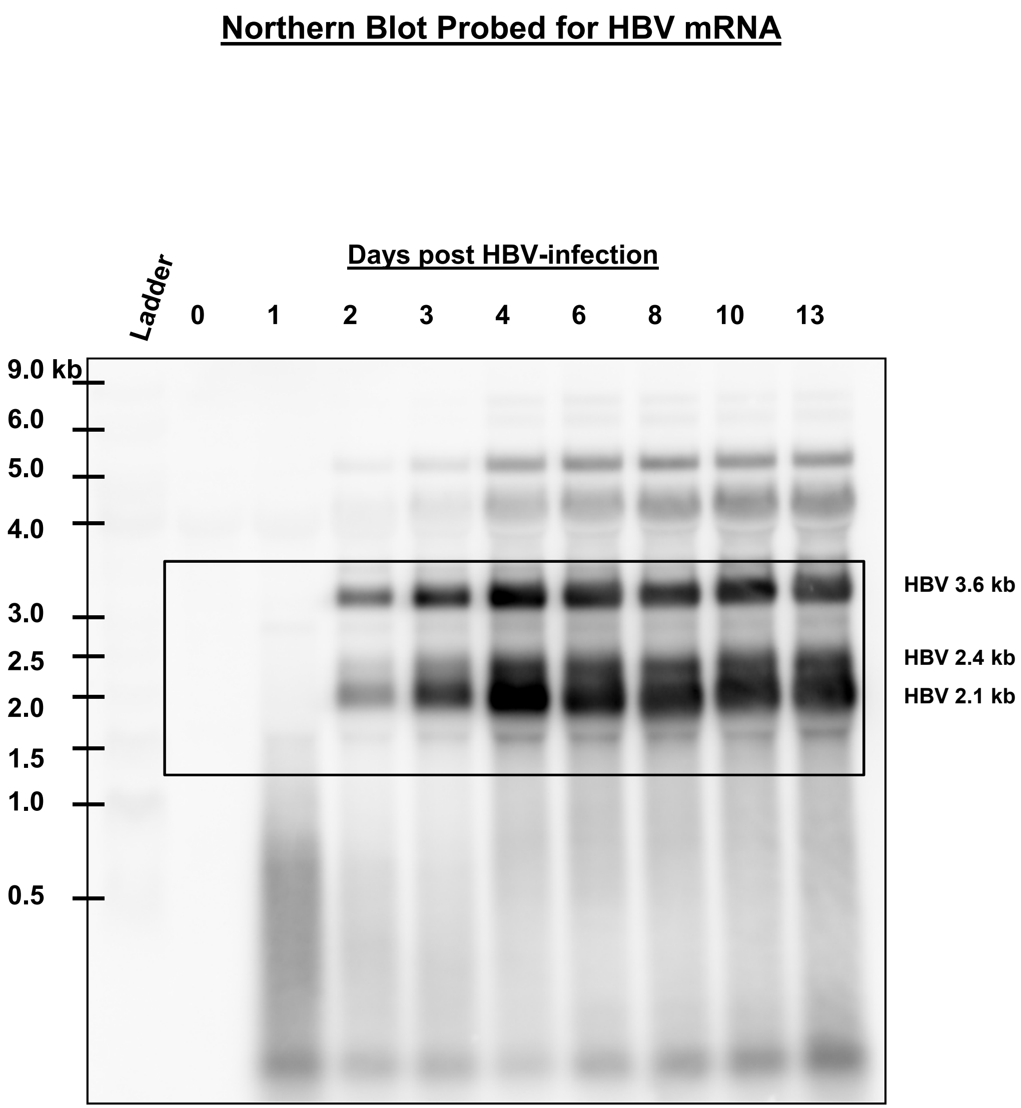

Fig. 3b. Northern blot (continued)

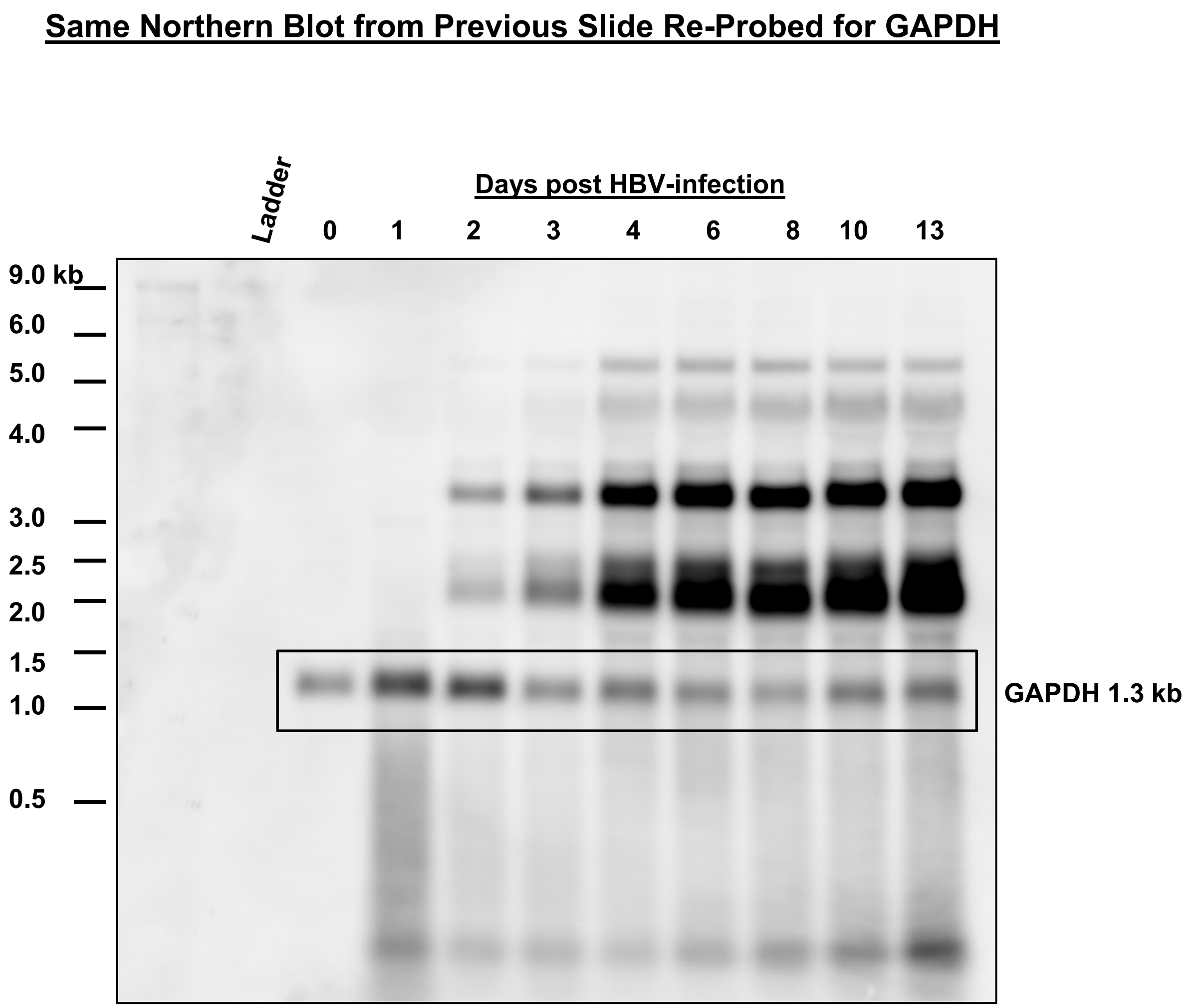

Supplementary Fig. 2a. Western blots

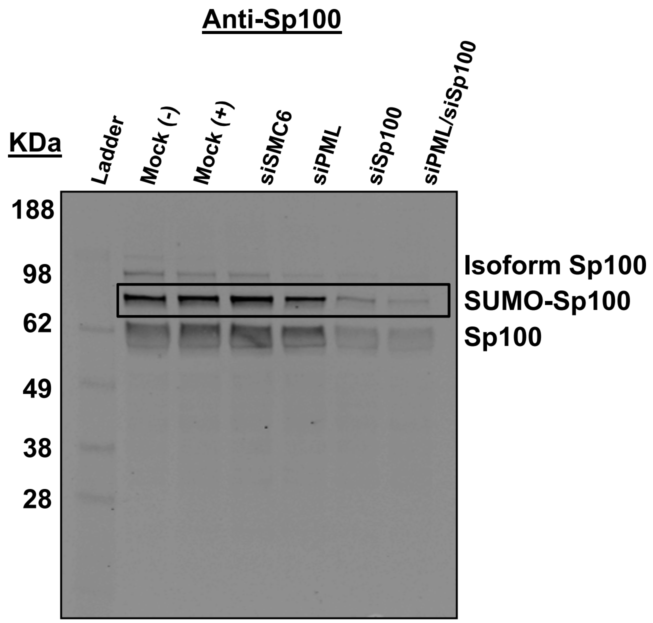

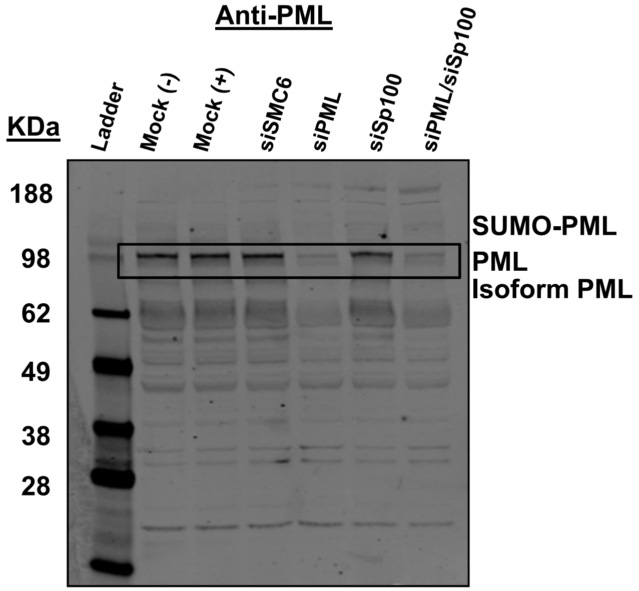

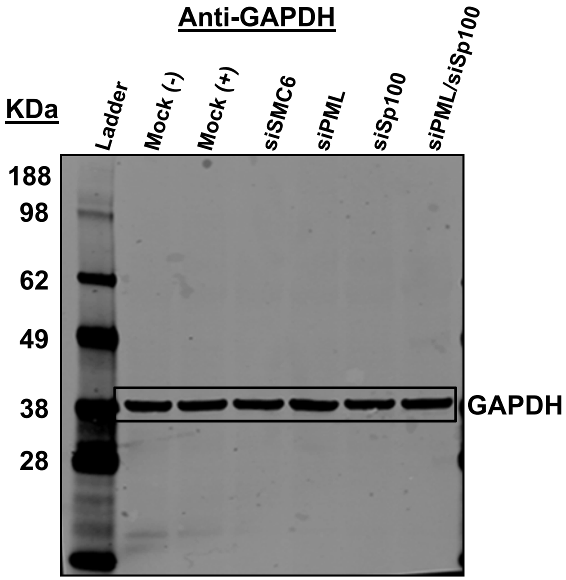

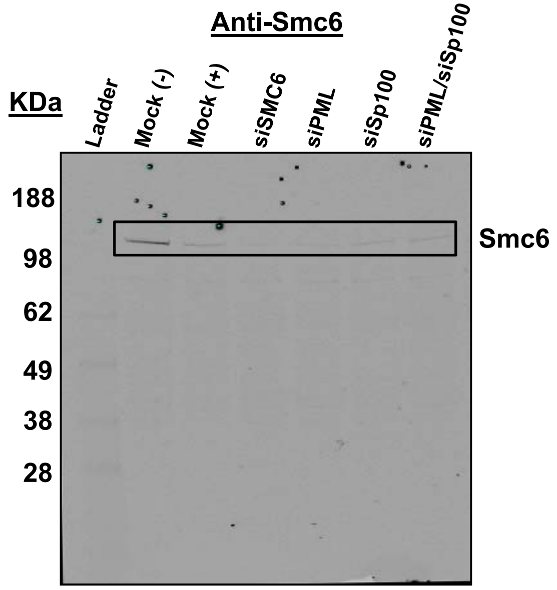

Supplementary Fig. 2b. Western blots

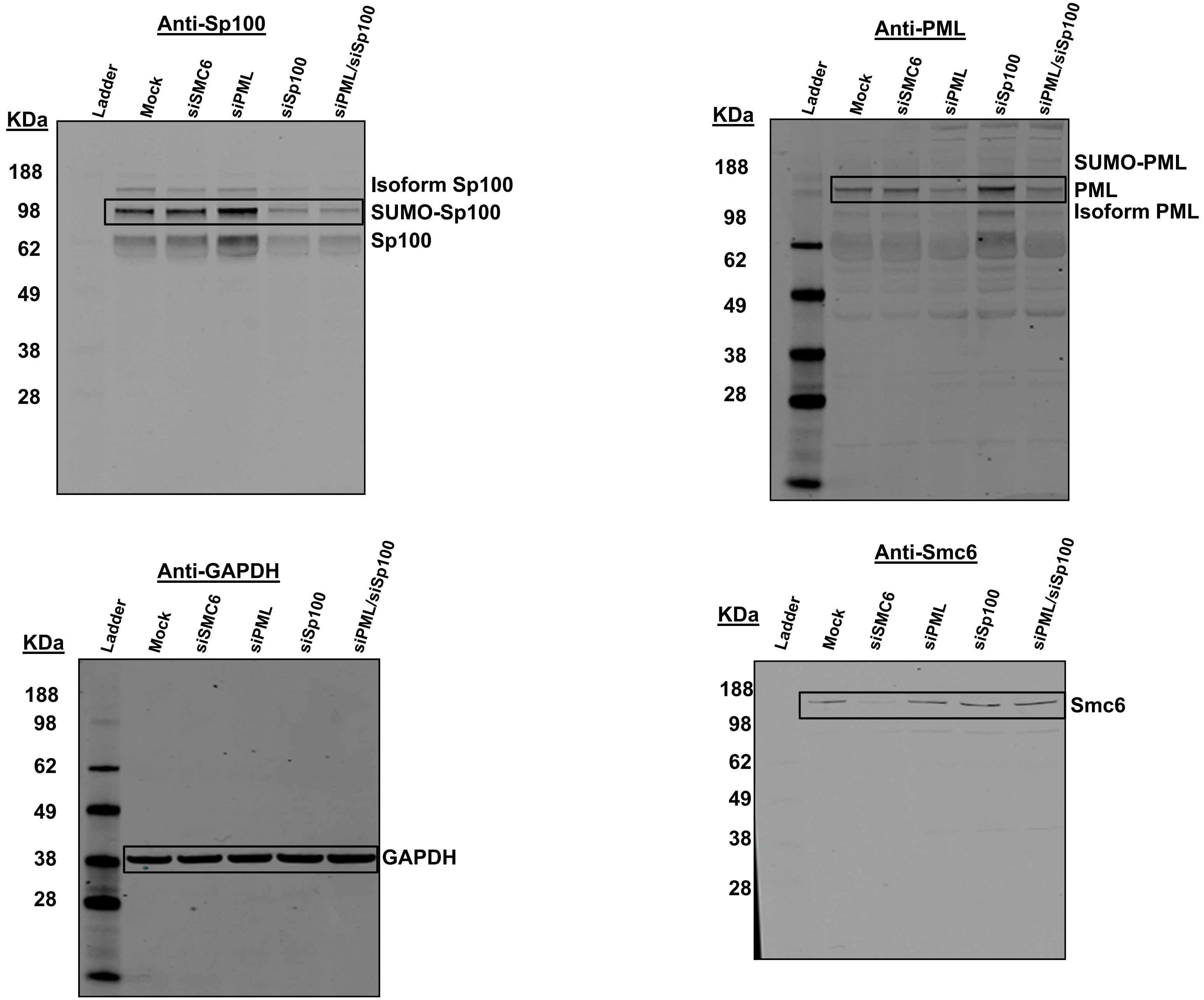

Supplementary Fig. 3. Northern blot

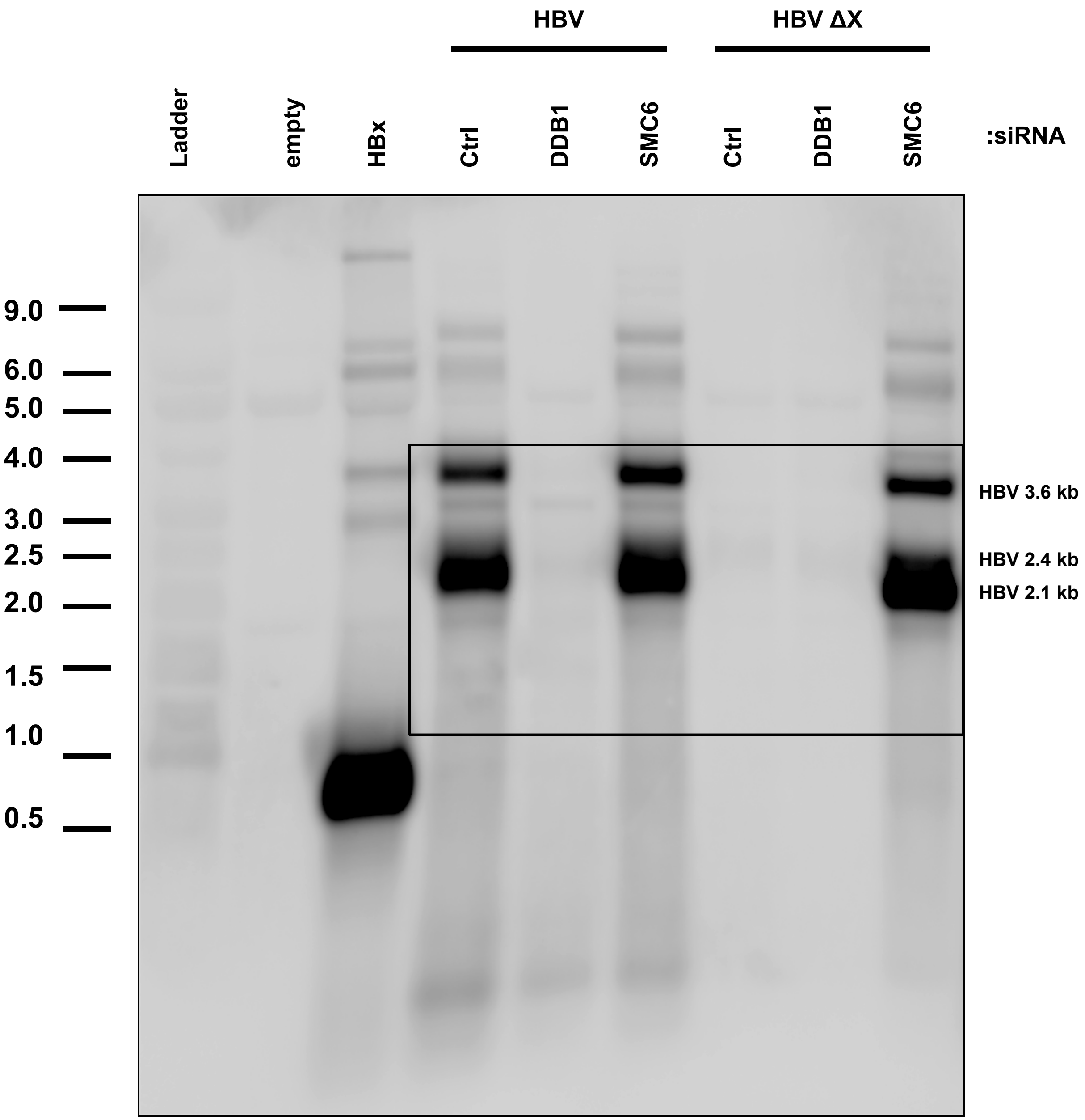

Supplementary Fig. 3. Northern blot (continued)

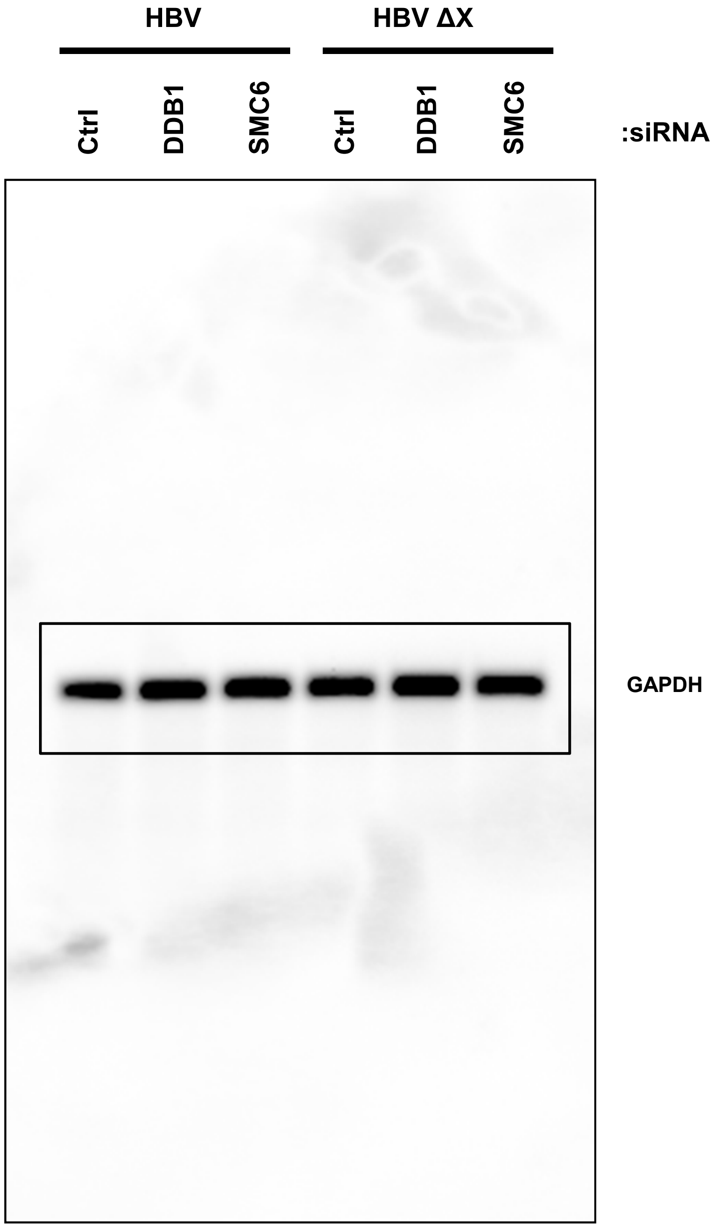

Supplement: S16 Fig — (PDF) [file pone.0169648.s016.pdf]
