# Supplementary material for: The Smc5/6 Complex Restricts HBV when Localized to ND10 without Inducing an Innate Immune Response and Is Counteracted by the HBV X Protein Shortly after Infection
Source: PLoS One. 2017 Jan 17;12(1):e0169648. doi: 10.1371/journal.pone.0169648 (PMC5240991; doi:10.1371/journal.pone.0169648)
Supplement: S3 Table — Cytokines from PHH infected with HBV or mock-infected, or treated with either IFN-α or poly(I:C). aMaximum cytokines levels at any time-point between 4h to 13d post-infection. bCytokine levels in mock-infected PHH were from time-matched samples. cMaximum cytokines levels in uninfected PHH at any time-point between 4h to 24h post-treatment. LLOQ; lower limit of quantitation. ULOQ; upper limit of quantitation. (DOC) [file pone.0169648.s019.doc]

**S3 Table. HBV infection does not induce cytokines in PHH (donor 1).**

| Cytokine | LLOQ (pg/mL) | Maximum cytokine level | | | |
| --- | --- | --- | --- | --- | --- |
|  |  | HBV-  infected^a^ | Mock-  infected^b^ | IFN-α^c^ | Poly(I:C)^c^ |
| IFN-α | 0.15 | <LLOQ | <LLOQ | 388.83 | <LLOQ |
| IFN-β | 2.73 | 6.62 | 6.62 | 6.62 | 6.62 |
| IFN-γ | 7.75 | <LLOQ | <LLOQ | <LLOQ | <LLOQ |
| IFN-λ1 | 13.90 | <LLOQ | <LLOQ | <LLOQ | 508.93 |
| IL-1α | 0.23 | <LLOQ | <LLOQ | <LLOQ | <LLOQ |
| IL-1β | 0.47 | <LLOQ | <LLOQ | <LLOQ | <LLOQ |
| IL-1RA | 33.66 | 1293.87 | 826.63 | 307.78 | 700.42 |
| IL-2 | 4.10 | <LLOQ | <LLOQ | <LLOQ | <LLOQ |
| IL-4 | 3.19 | <LLOQ | <LLOQ | <LLOQ | <LLOQ |
| IL-5 | 4.31 | <LLOQ | <LLOQ | <LLOQ | <LLOQ |
| IL-6 | 1.67 | <LLOQ | <LLOQ | <LLOQ | 2.79 |
| IL-7 | 0.19 | 7.24 | <LLOQ | 2.16 | 3.96 |
| IL-8 | 0.69 | 4882.58 | 2482.88 | 205.80 | 8771.87 |
| IL-9 | 6.98 | <LLOQ | <LLOQ | <LLOQ | <LLOQ |
| IL-10 | 0.86 | <LLOQ | <LLOQ | <LLOQ | <LLOQ |
| IL-12p70 | 1.49 | <LLOQ | <LLOQ | <LLOQ | <LLOQ |
| IL-13 | 0.74 | <LLOQ | <LLOQ | <LLOQ | <LLOQ |
| IL-15 | 4.25 | <LLOQ | <LLOQ | <LLOQ | <LLOQ |
| IL-17A | 3.18 | <LLOQ | <LLOQ | <LLOQ | <LLOQ |
| IL-18 | 2.50 | <LLOQ | 4.13 | <LLOQ | 5.50 |
| IL-21 | 1.61 | <LLOQ | <LLOQ | <LLOQ | <LLOQ |
| IL-22 | 25.20 | <LLOQ | 481.68 | <LLOQ | 40.51 |
| IL-23 | 1.47 | <LLOQ | <LLOQ | <LLOQ | <LLOQ |
| IL-27 | 4.65 | <LLOQ | <LLOQ | <LLOQ | <LLOQ |
| IL-31 | 33.80 | <LLOQ | <LLOQ | <LLOQ | <LLOQ |
| TNF-α | 1.69 | <LLOQ | <LLOQ | <LLOQ | <LLOQ |
| TNF-β | 1.24 | <LLOQ | <LLOQ | <LLOQ | <LLOQ |
| MCP-1 | 0.33 | 20.85 | 18.34 | 67.54 | 156.01 |
| MIP-1α | 0.56 | 4.92 | 8.26 | 13.93 | 148.49 |
| MIP-1β | 1.50 | 256.37 | 165.56 | 58.07 | 1392.20 |
| RANTES | 0.50 | 5.84 | 4.43 | 10.83 | 115.10 |
| Eotaxin | 0.80 | <LLOQ | 1.24 | 0.96 | 3.00 |
| GRO-α | 1.02 | 411.93 | 240.33 | 25.88 | 770.67 |
| IP-10 | 0.31 | 565.77 | 464.80 | >ULOQ | >ULOQ |
| SDF-1α | 4.50 | 84.36 | 63.34 | 72.44 | 341.36 |
| GM-CSF | 3.54 | <LLOQ | <LLOQ | <LLOQ | <LLOQ |
