# Supplementary material for: The Smc5/6 Complex Restricts HBV when Localized to ND10 without Inducing an Innate Immune Response and Is Counteracted by the HBV X Protein Shortly after Infection
Source: PLoS One. 2017 Jan 17;12(1):e0169648. doi: 10.1371/journal.pone.0169648 (PMC5240991; doi:10.1371/journal.pone.0169648)
Supplement: S4 Table — Cytokines from PHH infected with HBV or mock-infected, or treated with either IFN-α or poly(I:C). aMaximum cytokines levels at any time-point between 4h to 13d post-infection. bCytokine levels in mock-infected PHH were from time-matched samples. cMaximum cytokines levels in uninfected PHH at any time-point between 4h to 24h post-treatment. LLOQ; lower limit of quantitation. ULOQ; upper limit of quantitation. (DOC) [file pone.0169648.s020.doc]

**S4 Table. HBV infection does not induce cytokines in PHH (donor 2).**

| Cytokine | LLOQ (pg/mL) | Maximum cytokine level | | | |
| --- | --- | --- | --- | --- | --- |
|  |  | HBV-  infected^a^ | Mock-  infected^b^ | IFN-α^c^ | Poly(I:C)^c^ |
| IFN-α | 0.15 | <LLOQ | <LLOQ | 358.20 | <LLOQ |
| IFN-β | 2.73 | 27.11 | 18.77 | 25.01 | 10.61 |
| IFN-γ | 7.75 | <LLOQ | <LLOQ | <LLOQ | <LLOQ |
| IFN-λ1 | 13.90 | <LLOQ | <LLOQ | <LLOQ | 1482.38 |
| IL-1α | 0.23 | <LLOQ | <LLOQ | <LLOQ | <LLOQ |
| IL-1β | 0.47 | <LLOQ | <LLOQ | <LLOQ | <LLOQ |
| IL-1RA | 33.66 | 592.35 | 331.65 | 809.37 | 2061.26 |
| IL-2 | 4.10 | <LLOQ | <LLOQ | <LLOQ | <LLOQ |
| IL-4 | 3.19 | <LLOQ | <LLOQ | <LLOQ | <LLOQ |
| IL-5 | 4.31 | <LLOQ | <LLOQ | <LLOQ | <LLOQ |
| IL-6 | 1.67 | 1.95 | 1.81 | <LLOQ | 75.18 |
| IL-7 | 0.19 | 6.42 | 5.02 | 3.44 | 2.62 |
| IL-8 | 0.69 | 4474.49 | 9168.44 | 356.94 | 5923.84 |
| IL-9 | 6.98 | <LLOQ | <LLOQ | <LLOQ | <LLOQ |
| IL-10 | 0.86 | <LLOQ | <LLOQ | <LLOQ | <LLOQ |
| IL-12p70 | 1.49 | <LLOQ | <LLOQ | <LLOQ | <LLOQ |
| IL-13 | 0.74 | <LLOQ | <LLOQ | <LLOQ | <LLOQ |
| IL-15 | 4.25 | <LLOQ | <LLOQ | <LLOQ | <LLOQ |
| IL-17A | 3.18 | <LLOQ | <LLOQ | <LLOQ | <LLOQ |
| IL-18 | 2.50 | 4.61 | 13.39 | 4.91 | 4.13 |
| IL-21 | 1.61 | <LLOQ | <LLOQ | <LLOQ | <LLOQ |
| IL-22 | 25.20 | 255.78 | <LLOQ | 51.60 | 30.94 |
| IL-23 | 1.47 | <LLOQ | 7.57 | <LLOQ | 2.20 |
| IL-27 | 4.65 | <LLOQ | <LLOQ | <LLOQ | <LLOQ |
| IL-31 | 33.80 | <LLOQ | <LLOQ | <LLOQ | <LLOQ |
| TNF-α | 1.69 | <LLOQ | <LLOQ | <LLOQ | <LLOQ |
| TNF-β | 1.24 | <LLOQ | <LLOQ | <LLOQ | <LLOQ |
| MCP-1 | 0.33 | 73.11 | 47.81 | 178.79 | 242.36 |
| MIP-1α | 0.56 | 18.63 | 29.97 | 35.17 | 588.73 |
| MIP-1β | 1.50 | 132.44 | 279.84 | 185.23 | 1482.01 |
| RANTES | 0.50 | 5.09 | 9.46 | 17.05 | 146.16 |
| Eotaxin | 0.80 | <LLOQ | 1.48 | 1.36 | 3.23 |
| GRO-α | 1.02 | 84.02 | 207.77 | 26.17 | 481.84 |
| IP-10 | 0.31 | 317.29 | 463.25 | >ULOQ | >ULOQ |
| SDF-1α | 4.50 | 95.77 | 271.23 | 158.22 | 584.34 |
| GM-CSF | 3.54 | <LLOQ | <LLOQ | <LLOQ | <LLOQ |
