# Supplementary material for: The Smc5/6 Complex Restricts HBV when Localized to ND10 without Inducing an Innate Immune Response and Is Counteracted by the HBV X Protein Shortly after Infection
Source: PLoS One. 2017 Jan 17;12(1):e0169648. doi: 10.1371/journal.pone.0169648 (PMC5240991; doi:10.1371/journal.pone.0169648)
Supplement: S5 Table — aMaximum cytokines levels at any time-point between 4h to 13d post-infection. bCytokine levels in mock-infected PHH were from time-matched samples. cMaximum cytokines levels at any time-point between 4h to 24h post-treatment. LLOQ; lower limit of quantitation. ULOQ; upper limit of quantitation. (DOC) [file pone.0169648.s021.doc]

**S5 Table. HBVΔX infection does not induce cytokines in PHH.**

| Cytokine | LLOQ (pg/mL) | Maximum cytokine level | | | | | | |
| --- | --- | --- | --- | --- | --- | --- | --- | --- |
|  |  | HBVΔX-infected^a^ | | |  | Mock-  infected^b^ |  | Uninfected^c^ |
|  |  | No siRNA | siCtrl | siSmc6 |  | No siRNA |  | IFN-α |
| IFN-α | 0.21 | 0.5 | <LLOQ | 0.58 |  | 0.25 |  | 889.34 |
| IFN-β | 2.71 | <LLOQ | <LLOQ | <LLOQ |  | <LLOQ |  | <LLOQ |
| IFN-γ | 2.72 | <LLOQ | <LLOQ | <LLOQ |  | <LLOQ |  | <LLOQ |
| IFN-λ1 | 16.61 | <LLOQ | <LLOQ | <LLOQ |  | <LLOQ |  | <LLOQ |
| IL-1α | 0.31 | 3.01 | 0.55 | 5.53 |  | 3.12 |  | 10.73 |
| IL-1β | 0.56 | <LLOQ | <LLOQ | <LLOQ |  | <LLOQ |  | <LLOQ |
| IL-1RA | 10.66 | 4814.28 | 4195.29 | 3893.2 |  | 5720.92 |  | 95522.27 |
| IL-2 | 4.29 | <LLOQ | <LLOQ | <LLOQ |  | <LLOQ |  | <LLOQ |
| IL-4 | 2.99 | <LLOQ | <LLOQ | <LLOQ |  | <LLOQ |  | <LLOQ |
| IL-5 | 1.80 | <LLOQ | <LLOQ | <LLOQ |  | <LLOQ |  | <LLOQ |
| IL-6 | 2.55 | <LLOQ | <LLOQ | <LLOQ |  | <LLOQ |  | <LLOQ |
| IL-7 | 0.82 | 15.33 | 15.15 | 15.38 |  | 2.47 |  | 5.25 |
| IL-8 | 2.76 | 1924.48 | 1086.25 | 3267.51 |  | 1987.53 |  | 692.48 |
| IL-9 | 9.53 | 4.86 | <LLOQ | 12.91 |  | 12.91 |  | <LLOQ |
| IL-10 | 2.85 | <LLOQ | <LLOQ | <LLOQ |  | <LLOQ |  | <LLOQ |
| IL-12p70 | 1.69 | <LLOQ | <LLOQ | <LLOQ |  | <LLOQ |  | <LLOQ |
| IL-13 | 0.61 | <LLOQ | <LLOQ | <LLOQ |  | <LLOQ |  | <LLOQ |
| IL-15 | 5.98 | <LLOQ | <LLOQ | <LLOQ |  | <LLOQ |  | 78.93 |
| IL-17A | 0.56 | <LLOQ | <LLOQ | <LLOQ |  | <LLOQ |  | <LLOQ |
| IL-18 | 2.32 | <LLOQ | <LLOQ | <LLOQ |  | <LLOQ |  | <LLOQ |
| IL-21 | 2.63 | <LLOQ | <LLOQ | <LLOQ |  | <LLOQ |  | <LLOQ |
| IL-22 | 34.53 | <LLOQ | <LLOQ | <LLOQ |  | <LLOQ |  | <LLOQ |
| IL-23 | 4.12 | <LLOQ | <LLOQ | <LLOQ |  | <LLOQ |  | <LLOQ |
| IL-27 | 19.63 | <LLOQ | <LLOQ | <LLOQ |  | <LLOQ |  | <LLOQ |
| IL-31 | 10.92 | <LLOQ | <LLOQ | <LLOQ |  | <LLOQ |  | 79.62 |
| TNF-α | 6.84 | <LLOQ | <LLOQ | <LLOQ |  | <LLOQ |  | <LLOQ |
| TNF-β | 5.31 | <LLOQ | <LLOQ | <LLOQ |  | <LLOQ |  | 26.78 |
| MCP-1 | 0.32 | 17.66 | 20.14 | 26.47 |  | 19.63 |  | 281.59 |
| MIP-1α | 0.55 | 5.48 | 5.85 | 8.31 |  | 3.63 |  | 8.58 |
| MIP-1β | 5.59 | 10.63 | 11.87 | 14.77 |  | 11.26 |  | 24.11 |
| RANTES | 0.49 | 3.43 | 5.23 | 5.89 |  | 4.28 |  | 21.36 |
| Eotaxin | 0.21 | 1.09 | 0.75 | 1.02 |  | 1.43 |  | 0.97 |
| GRO-α | 3.35 | 211.86 | 86.08 | 270.37 |  | 141.8 |  | 68.33 |
| IP-10 | 0.36 | 261.19 | 223.83 | 208.67 |  | 320.35 |  | >ULOQ |
| SDF-1α | 4.73 | 58.36 | 18.21 | 76.64 |  | 55.15 |  | 87.89 |
| GM-CSF | 17.35 | <LLOQ | <LLOQ | <LLOQ |  | <LLOQ |  | <LLOQ |
