# Supplementary material for: The Smc5/6 Complex Restricts HBV when Localized to ND10 without Inducing an Innate Immune Response and Is Counteracted by the HBV X Protein Shortly after Infection
Source: PLoS One. 2017 Jan 17;12(1):e0169648. doi: 10.1371/journal.pone.0169648 (PMC5240991; doi:10.1371/journal.pone.0169648)
Supplement: S6 Table — In addition to the manufacturer's validation, the specificity of the primary antibodies for microscopy and Western blot applications was confirmed by siRNA and/or over-expression analysis. CM: confocal microscopy, Epi: epifluorescence microscopy, WB: Western blot, FC: flow cytometry, n/a: not applicable. (DOC) [file pone.0169648.s022.doc]

**S6 Table. Antibody summary.**

| Host animal | Clonality | Reactivity | Antigen | Clone ID | Conjugate | Company | Cat. No. | Antibody Registry No. | Application |
| --- | --- | --- | --- | --- | --- | --- | --- | --- | --- |
| Mouse | Monoclonal | Human | Smc6 | 2E7 | n/a | Abgent | AT3956a | AB_1554548 | CM, Epi, WB |
| Rabbit | Polyclonal | Human | Smc5 | - | n/a | Bethyl Laboratories | A300-236A | AB_2192785 | CM |
| Rabbit | Polyclonal | Human | PML | - | n/a | Abcam | ab53773 | AB_882092 | CM, Epi, WB |
| Rabbit | Polyclonal | HBV | HBV core | - | n/a | Dako | B0586 | AB_2335704 | CM, Epi |
| Mouse | Monoclonal | Human | PML | PG-M3 | n/a | Santa Cruz | sc-966 | AB_2252139 | CM |
| Rabbit | Polyclonal | HBV | HBsAg | - | n/a | Virostat | 1811 | n/a | CM |
| Rabbit | Polyclonal | Human | Sp100 | - | n/a | Novus Biologicals | NBP1 89457 | AB_11036138 | CM, WB |
| Rabbit | Monoclonal | Human | GAPDH | 14C10 | n/a | Cell Signaling | 2118 | AB_10698756 | WB |
| Goat | Polyclonal | Rabbit | Rabbit IgG | - | Alexa Fluor-488 | Life Technologies | A11034 | AB_10562715 | CM |
| Goat | Polyclonal | Mouse | Mouse IgG | - | Alexa Fluor-488 | Life Technologies | A11029 | AB_2534088 | CM |
| Goat | Polyclonal | Rabbit | Rabbit IgG | - | Alexa Fluor-594 | Life Technologies | A11037 | AB_2534095 | CM |
| Goat | Polyclonal | Mouse | Mouse IgG | - | Alexa Fluor-594 | Life Technologies | A11032 | AB_2534091 | CM |
| Donkey | Polyclonal | Mouse | Mouse IgG | - | Alexa Fluor-488 | Life Technologies | A21202 | AB_2535788 | Epi |
| Donkey | Polyclonal | Rabbit | Rabbit IgG | - | Alexa Fluor-555 | Life Technologies | A31572 | AB_2536182 | Epi |
| Donkey | Polyclonal | Rabbit | Rabbit IgG | - | Alexa Fluor-594 | Life Technologies | A21207 | AB_141637 | Epi |
| Goat | Polyclonal | Mouse | Mouse IgG | - | IRDye 800CW | Li-Cor | 926-32210 | AB_621842 | WB |
| Goat | Polyclonal | Rabbit | Rabbit IgG | - | IRDye 680RD | Li-Cor | 926-68071 | AB_10956166 | WB |
| Mouse | Monoclonal | Human | CD81 | JS-81 | Allophycocyanin | BD Biosciences | 551112 | [AB_398491](http://antibodyregistry.org/AB_398491) | FC |
| Mouse | Monoclonal | Human | CD68 | Y1/82A | Fluorescein | BD Biosciences | 562117 | [AB_10896283](http://antibodyregistry.org/AB_10896283) | FC |
| Rabbit | Polyclonal | Human | Albumin | - | Fluorescein | Rockland | 600-402-033 | [AB_828087](http://antibodyregistry.org/AB_828087) | FC |
| Mouse | Monoclonal | Human | CD45 | 2D1 | Fluorescein | BD Biosciences | 340614 | [AB_400074](http://antibodyregistry.org/AB_400074) | FC |
| Mouse | Monoclonal | Human | CD299 | 16E7 | Allophycocyanin | eBioscience | 17-2999-42 | n/a | FC |
| Mouse | Monoclonal | Human | HLA-DR | LN3 | Fluorescein | eBioscience | 11-9956-42 | n/a | FC |
